# Use of novel haptens in the production of antibodies for the detection of tryptamines†

**DOI:** 10.1039/c8ra02528b

**Published:** 2018-05-01

**Authors:** Michal Maryška, Lucie Fojtíková, Radek Jurok, Barbora Holubová, Oldřich Lapčík, Martin Kuchař

**Affiliations:** Department of Chemistry of Natural Compounds, University of Chemistry and Technology Technická 5, 166 28 Praha 6 – Dejvice Czech Republic martin.kuchar@vscht.cz +420-220-444-422 +420-220-444-432; Department of Organic Chemistry, University of Chemistry and Technology Technická 5, 166 28 Praha 6 – Dejvice Czech Republic; Department of Biochemistry and Microbiology, University of Chemistry and Technology Technická 3, 166 28 Praha 6 – Dejvice Czech Republic; Forensic Laboratory of Biologically Active Substances, University of Chemistry and Technology Technická 3, 166 28 Praha 6 – Dejvice Czech Republic

## Abstract

Tryptamines are a group of hallucinogenic drugs whose detection in body fluids could be simplified by immunochemical assay kits. Antibodies for these assays are obtained by the immunization of laboratory animals with conjugates of a hapten similar to the target analyte and a suitable protein. Therefore we synthesized novel haptens derived from tryptamine-based drugs, with *N*,*N*-dimethyltryptamine (DMT), 5-methoxy-*N*,*N*-dimethyltryptamine (5-MeO-DMT) and *N*,*N*-diisopropyltryptamine (DiPT) selected as the target analytes. Their structures were modified with a short linker ended with a carboxylic group. The haptens were conjugated with bovine serum albumin (BSA) and rabbits were immunized with the conjugates. The obtained polyclonal antibodies showed good reactivity and the LOD of the constructed ELISAs was in the range 0.006–0.254 ng mL^−1^. Thus, they are suitable for the development of immunochemical assay kits.

## Abstract 2

We synthesized haptens derived from tryptamine-based drugs and used them in the production of antibodies against various tryptamines.

## Graphical abstract

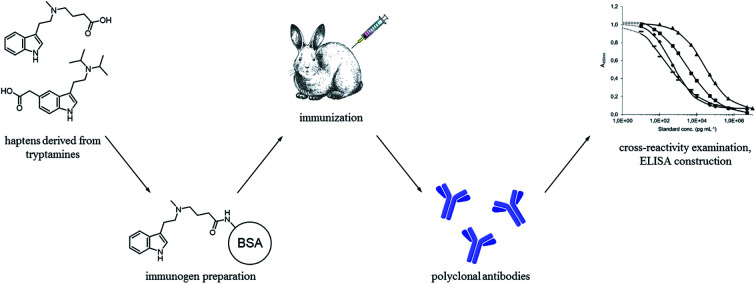

## 1. Introduction

Tryptamines, a group of hallucinogenic drugs derived from tryptamine, include both natural and synthetic compounds. The best known tryptamine is dimethyltryptamine (DMT), the active compound of ayahuasca, the ritual beverage, which has traditionally been used in several South American indigenous cultures.^1^ Other traditional natural tryptamines are psilocybin and psilocin, active compounds of the *Psilocybe* mushrooms also known as ‘magic mushrooms’. Over the past few decades, lots of synthetic derivatives of tryptamine have been prepared. Their rise in popularity has been attributed to Alexander Shulgin, whose book TiHKAL (Tryptamines I have known and loved) described the synthesis and effects of many of them.^2^ These synthetic tryptamines belong to the group of novel psychoactive substances (NPS), which are produced to give ‘legal highs’ and bypass legal restrictions.^3^ Although there is widespread use of NPS on the drug scene, options for their detection, including for the detection of tryptamines, remain rather limited.

There are currently only two methods for the detection of tryptamines. The first involves the use of specific colour tests, which are based on the reaction of specific reagents forming coloured products with indole-derived compounds. But, with so many different NPS now on the drug scene, colour tests are not reliable because of their low specificity.^4^ The more commonly used second method involves the analysis of tryptamines by liquid chromatography coupled with mass spectrometry (LC-MS).^5,6^ This method is precise, capable of determining multiple targets in one run, offers low detection limits and can be used for various matrices, especially body fluids. However, it is also demanding with respect to cost, sample preparation and analysis time. Furthermore, LC-MS-based techniques cannot be used in the field.

Because of the above-mentioned disadvantages, attention has focused on immunochemical methods, such as ELISA (enzyme-linked immunosorbent assay) and LFIA (lateral flow immunosorbent assay). Antibodies for immunochemical detection are usually produced by the immunization of laboratory animals. Because tryptamines are haptens, *i.e.* molecules too small to be immunogenic on their own, they must be conjugated to a carrier protein prior to immunization. To do this, molecules of the target analytes are modified with short linkers containing a suitable functional group. Using bufotenine and serotonin as haptens, Skerritt *et al.* reported the development of an ELISA kit for the detection of DMT and 5-MeO-DMT in *Phalaris* plants.^7^ However, this assay is limited only to these natural tryptamines and is not selective between them. Yamaguchi *et al.* prepared monoclonal antibodies against psilocin for identification of magic mushrooms.^8^ These antibodies showed cross reactivity with DMT, but their limit of detection was relatively high. Thus far, no other immunochemical assay has been reported that targets either natural or synthetic tryptamines.

The development of LFIA kits for the detection of synthetic and other tryptamines is needed to screen potential users. To provide such a kit, we first synthesized novel haptens carrying a short linker with a carboxyl group for the production of antibodies selective to different tryptamines. Rabbits were immunized with conjugates of these haptens with bovine serum albumin (BSA). The antibodies obtained from the immunization showed expedient sensitivity and selectivity for individual tryptamines, and, thus, appear to provide a viable basis for LFIA development.

## 2. Materials and methods

Ethyl 2-bromoacetate was obtained from Merck and *N*,*N*′-dicyclohexylcarbodiimide (DCC) was obtained from Fluka. Diisobutylaluminium hydride (DIBAL-H) solution in hexane, oxalyl chloride and dry DMF were purchased from Acros. Other reagents were purchased from Sigma-Aldrich. Dry THF, dichloromethane and diethyl ether were dried with molecular sieves. All reactions were carried out under argon atmosphere. Thin layer chromatography (TLC) was performed on aluminium backed sheets coated with 60F 254 silica gel from Merck. Column chromatography was performed on silica (0.045–0.200 μm) from Merck. Reverse phase chromatography was carried out using a CombiFlash Rf 200 apparatus (Teledyne ISCO) with prepacked Redisep Rf Gold C18 columns (packed with C18-reverse phase silica gel). NMR spectra were recorded on a Varian Gemini 300 (300 MHz for ^1^H; 75 MHz for ^13^C) or Agilent 400-MR DD2 (400 MHz for ^1^H; 100 MHz for ^13^C) spectrometers. High resolution mass spectra were measured on a LTQ Orbitrap XL (Thermo Fischer Scientific) spectrometer using ESI ionization technique. Mass spectra of hapten–protein conjugates were measured on a Bruker Autoflex Speed MALDI-TOF/TOF spectrometer. Microplate reader uQuant BIO-TEK was from Inc. Winooski, USA and 96-well polystyrene microtiter plates Costar 9018 were purchased from Corning Inc., USA. Peroxidase labelled goat anti-rabbit antibody (GAR-Po) was obtained from Nordic Immunological Laboratories, Netherlands. Analytical standards psilocin and psilocybin were obtained from THC Pharm GmbH The Health Concept, Germany. Analytical standards of DMT, 5-MeO-DMT, DiPT and 5-MeO-DiPT were prepared according to literature^9^ in purity ≥98% (LC). Serotonin was purchased from Sigma-Aldrich. Other tryptamines 5-MeO-DALT, 4-HO-MET, acetylpsilocin, αMT and 5-MeO-DIPT were generously donated by the Institute of Criminalistics Prague.

### 2.1 Preparation of conjugates

Following the procedure we described previously,^10^ the prepared haptens I–IV were conjugated to BSA using activated ester method. Obtained conjugates I–IV were analyzed by MALDI-TOF to determine the number of hapten molecules bound to the protein. The average values were calculated from the peak with highest intensity.

### 2.2 Immunisation and antiserum preparation

Four different immunisation conjugates (conjugate I–IV) were used. Antisera were produced in rabbits and obtained according to the procedure described previously.^11^ Stock solutions of antisera were prepared by dissolving 1 mg of lyophilisate in 1 ml of PBS and were stored at −20 °C.

### 2.3 Indirect competitive ELISA

ELISA microtiter plates were coated with the coating conjugates (appropriately diluted in the carbonate–bicarbonate buffer; 100 μl per well) and incubated at 4 °C overnight. The following day, the plates were washed with the PBS-Tw (4 times, 350 μl per well). The suitable standard (the concentration range of 0–500 ng mL^−1^ in the PBS; 50 μl per well) was added into microtiter plates followed by the solution of appropriate antiserum (diluted in the PBS-0.1% BSA (w/v); 50 μl per well) and incubated at room temperature for 1 hour. Microtiter plates were washed again (4 times, 350 μl per well) and GAR-Po (diluted 1 : 10 000 in the PBS-Tw; 100 μl per well) was added and incubated at room temperature for 1 h. After the washing step, the TMB substrate solution was added (100 μl per well) and incubated at room temperature for 10 min. The enzyme reaction was stopped by addition of 2 mol L^−1^ H_2_SO_4_ (50 μl per well) and the absorbance was measured at 450 nm.

### 2.4 Calibration standard curve

Sigmoid calibration standard curves were obtained by plotting the mean values of absorbance against the logarithm of standard concentrations through a four-parameter logistic equation as described previously.^11^ The limit of detection (LOD) was defined as the concentration of an analyte corresponding to the maximum assay signal minus 3× standard deviation (SD) in accordance with the calibration curve (the blank was calculated from 3 parallel determinations with the absence of an analyte). The IC_50_ corresponded to the concentration of analyte giving 50% inhibition of the asymptotic maximum. The linear working range corresponded to the analyte concentration causing the 20–80% inhibition of the maximum assay signal.

### 2.5 Specificity of antibodies

To verify the ability of the antibodies to react with similar epitopes on different tryptamines, the cross-reactivity (CR) tests were performed. The percent CR (CR (%)) was calculated from IC_50_ obtained from the calibration curves: (IC_50_ of target drug)/(IC_50_ of tested compound) × 100.

## 3. Results and discussion

**Fig. 1:**
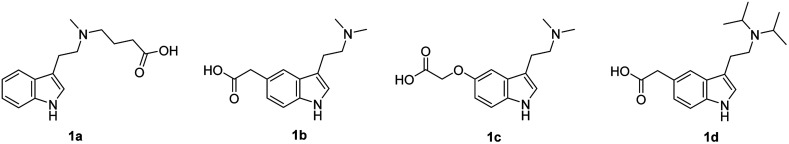
Designed haptens I–IV.

To design the structure of haptens, DMT, 5-MeO-DMT and DiPT were selected as target analytes and their structures were modified with a short alkyl chain ended with a carboxylic group. Four different haptens were prepared, differing in a position of the linker (Fig. 1). Hapten I (1a) with the linker on the amino group was derived from DMT. Haptens II–IV (1b–d) with the linker in position 5 of indole ring were derived from 5-MeO-DMT (haptens II and III) and DiPT, respectively (hapten IV).

**Fig. 2:**
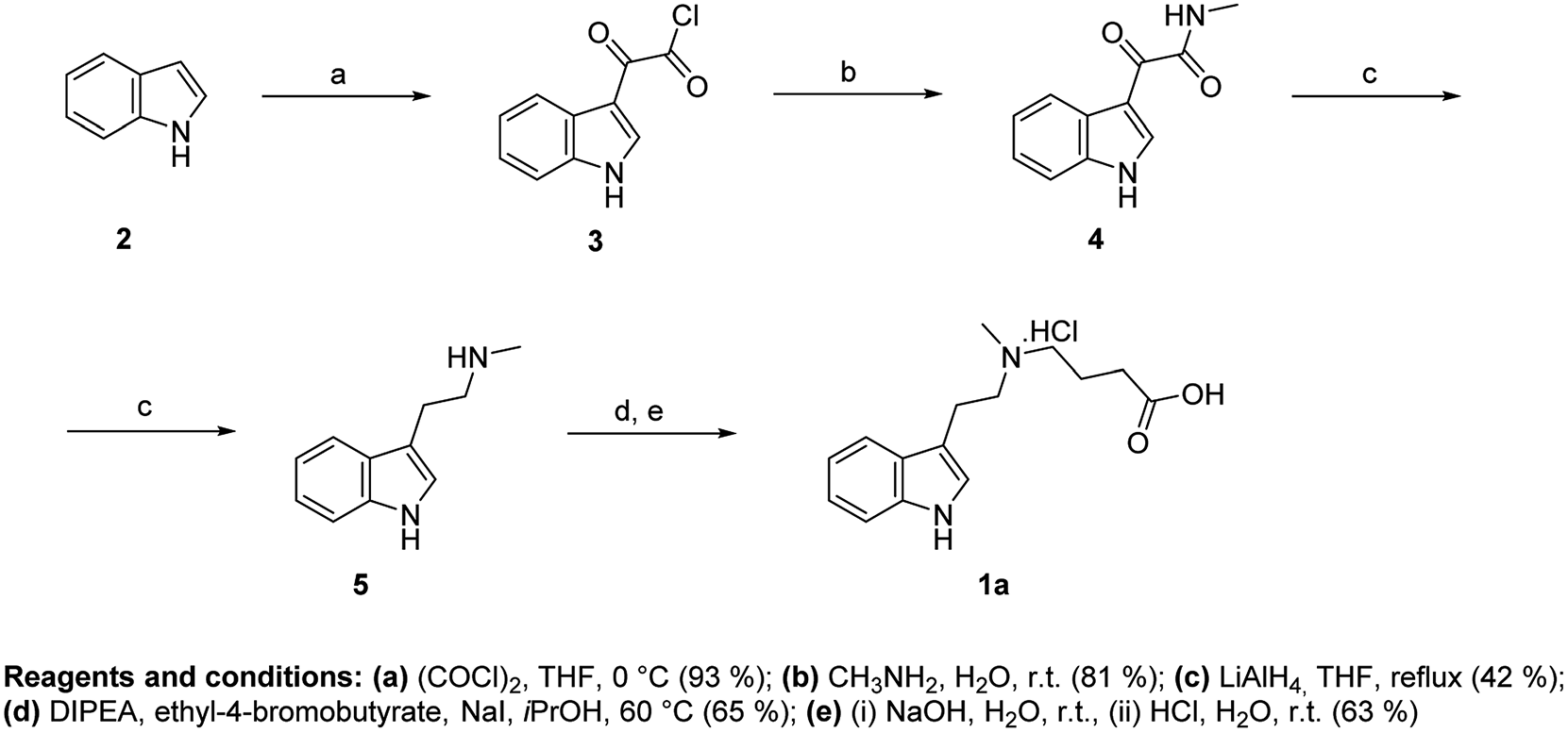
Synthesis of hapten I.

Synthesis of hapten I (1a) started from indole (2) and used a sequence of reactions developed by Speeter and Anthony to obtain *N*-methyltryptamine (5).^9,12^ Alkylation of 5 with ethyl-4-bromobutyrate and subsequent hydrolysis of ester 6 yielded hapten I (1a) (Fig. 2).

**Fig. 3:**
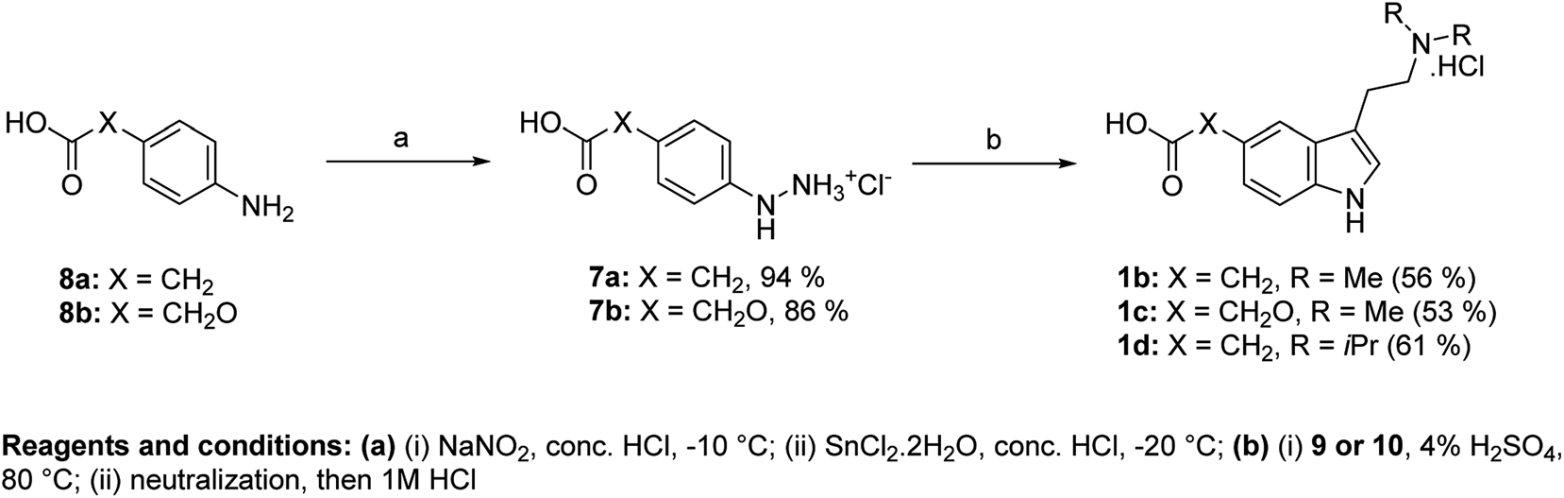
Direct synthesis of haptens II–IV using Fischer reaction.

We used an approach based on the Fischer reaction for the synthesis of haptens II–IV (1b–d). Haptens were obtained directly from suitable arylhydrazines and amino acetals (Fig. 3). Arylhydrazinium chlorides 7a,b were prepared in high yields from aniline derivatives 8a,b by diazotation and subsequent reduction of diazonium salts with SnCl_2_·2H_2_O.^13^

**Fig. 4:**
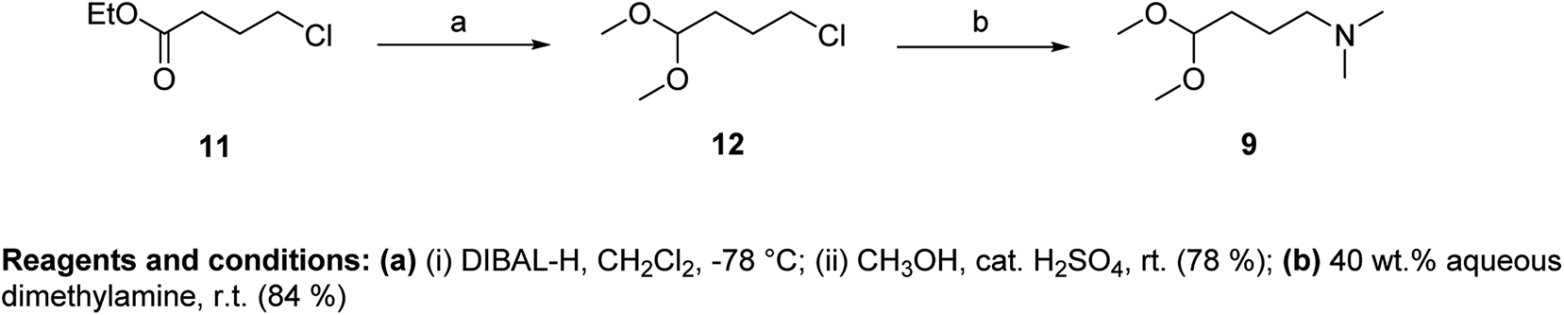
Preparation of *N*,*N*-dimethylamino-1,1-dimethoxybutane (9).

As an acetal component of the Fischer reaction, we used *N*,*N*-dimethylamino-1,1-dimethoxybutane (9) and *N*,*N*-diisopropylamino-1,1-dimethoxybutane (10). Compound 9 was prepared in two steps from ethyl-4-chlorobutyrate (11) (Fig. 4).

**Fig. 5:**
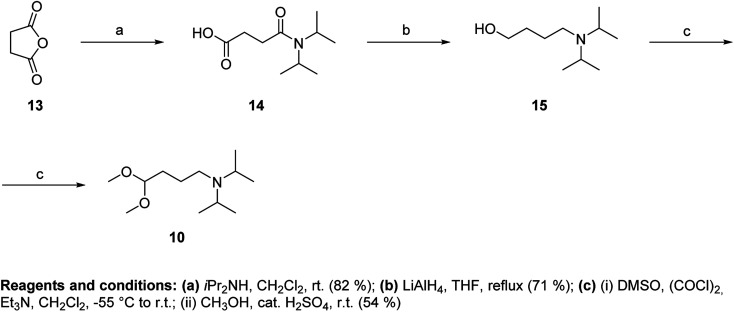
Synthesis of *N*,*N*-diisopropylamino-1,1-dimethoxybutane (10).

Because the same sequence of reactions could not be used in the synthesis of acetal 10, we developed a different approach starting from succinic anhydride (13) (Fig. 5). Reaction of 13 with diisopropylamine and subsequent reduction of acid 14 with LiAlH_4_ led to the amino alcohol 15. Swern conditions were used for oxidation of 15 to aldehyde, which was immediately acetalized to obtain 10 in moderate yield.

Haptens I–IV (1a–d) were conjugated with bovine serum albumin (BSA) using the methodology previously employed in our group.^10,14^ Conjugates I–IV were submitted to MALDI-TOF analysis and the number of hapten molecules was determined as follows: 13 for conjugate I, 31 for conjugate II, 28 for conjugate III and 37 for conjugate IV.

**Fig. 6:**
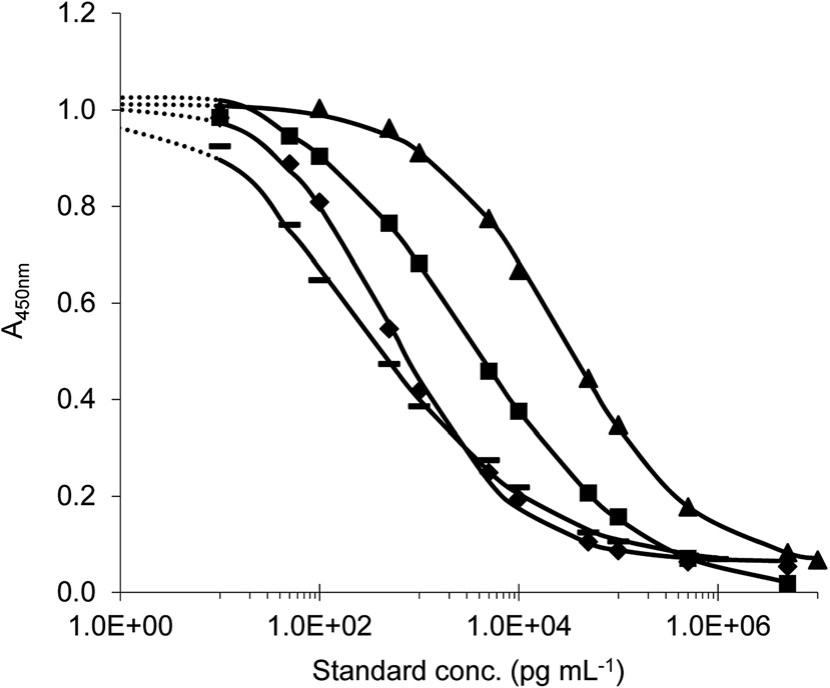
Typical ELISA standard curves (◆) ELISA I (standard DMT); (▲) ELISA II (standard 5-MeO-DMT); (▬) ELISA III (standard 5-MeO-DMT); (■) ELISA IV (standard DiPT).

Antisera were collected by immunization of rabbits with all of prepared conjugates.^11^ The indirect competitive format of ELISA was used for antiserum testing. First, checkerboard titrations were performed and suitable immunoreagent concentrations were determined when the maximum absorbance ranged from 1.2 to 1.9. Then the calibration curves with target analyte were constructed. The antibody with the highest sensitivity to the appropriate analyte was selected (on the basis of the lowest IC_50_ values) for each immunisation conjugate and further tests were made. In this study, four different ELISAs (ELISA I–IV) for the detection of tryptamine-based drugs were successfully developed. Titration standard curves are shown in Fig. 6 and analytical parameters of methods are summarized in the table (Table 1).

**Table 1:** Analytical parameters of assembled ELISAs

	IC_50_^a^ ± SD^b^ (ng ml^−1^)	Linear working range (ng ml^−1^)	LOD^c^ ± SD^b^ (ng ml^−1^)
ELISA I	0.52 ± 0.17	0.11–4.24	0.017 ± 0.005
ELISA II	27.3 ± 5.20	4.08–241.96	0.254 ± 0.059
ELISA III	0.29 ± 0.07	0.03–4.93	0.006 ± 0.002
ELISA IV	3.36 ± 0.28	0.27–45.13	0.034 ± 0.005

a IC_50_, 50% intercept.

b SD, standard deviation.

c LOD, limit of detection.

The specificity of obtained antibodies was evaluated with 102 new psychoactive substances and non-hallucinogenic compounds: 11 synthetic or natural tryptamines, 18 phenylethylamines, 13 piperazines, 18 synthetic cannabinoids, 27 cathinones, 16 non-hallucinogenic substance. Cross-reactivity values for tryptamines are summarized in Table 2. Positive cross-reactivity values were obtained only for synthetic tryptamines and psilocin. No cross-reactivity was observed with other tested compounds. Our results show that synthesized haptens and immunogens are functional and that they can be used for the production of antibodies. These antibodies can be applied in the development of immunoanalytical methods for the tryptamine-based drug detection.

**Table 2:** Cross-reactivity data for developed ELISA^a^

Compound	Cross-reactivity (%)			
	ELISA I	ELISA II	ELISA III	ELISA IV
DMT	100.00	53.48	38.89	0.09
5-MeO-DMT	0.57	100.00	100.00	0.04
5-MeO-DALT	1.90	0.95	2.46	0.19
DiPT	2.00	0.09	0.21	100.00
4-HO-MET	2.16	5.87	20.08	0.05
αMT	0.14	0.07	0.04	<0.001
*O*-Acetylpsilocin	0.06	2.81	23.04	<0.001
Psilocin	3.60	225.71	149.63	0.10
Psilocybin	<0.001	0.07	0.08	<0.001
Serotonin	<0.001	<0.001	<0.001	<0.001
5-MeO-DiPT	0.01	<0.001	0.43	88.43

a DMT: *N*,*N*-dimethyltryptamine, 5-MeO-DMT: 5-methoxy-*N*,*N*-dimethyltryptamine, 5-MeO-DALT: *N*,*N*-diallyl-5-methoxytryptamine, DiPT: *N*,*N*-diisopropyltryptamine, 4-HO-MET: 4-hydroxy-*N*-methyl-*N*-ethyltryptamine, αMT: α-methyltryptamine, 5-MeO-DiPT: 5-methoxy-*N*,*N*-diisopropyltryptamine.

## 4. Experimental

### 4.1 Synthesis of haptens

#### 4.1.1 Hapten I

##### 2-(1*H*-indol-3-yl)-2-oxoacetyl chloride (3)

Compound 3 was prepared according to literature^9^ from indole (2) (2.00 g, 17.1 mmol) as a yellow solid (3.31 g, 93%).

##### 2-(1*H*-indol-3-yl)-*N*-methyl-2-oxoacetamide (4)

Glyoxylyl chloride 3 (3.21 g, 15.5 mmol) was added in portions to an ice cold aqueous solution of methylamine (20 ml, 40 wt%) and resulting suspension was stirred at 0 °C for 2 hours. The precipitate was filtered and washed with water and diethyl ether. Recrystallization of the crude product from THF/Et_2_O gave the titled compound 4 as a pale yellow solid (2.53 g, 81%). Mp = 214–215 °C (lit.^15^ 218–219 °C); ^1^H NMR (300 MHz, DMSO-d_6_) *δ*: 2.75 (d, 3H, *J* = 4.7 Hz), 7.22–7.30 (m, 2H), 7.50–7.57 (m, 1H), 8.19–8.26 (m, 1H), 8.68 (m br, 1H), 8.77 (s, 1H), 12.21 (s br, 1H); ^13^C NMR (75 MHz, DMSO-d_6_) *δ*: 25.57, 112.19, 112.56, 121.33, 122.53, 123.42, 126.25, 136.27, 138.54, 164.09, 182.08; HRMS (ESI): *m*/*z* [M + Na]^+^ calculated for C_11_H_10_N_2_O_2_: 225.06345, found 225.06329.

##### *N*-methyltryptamine (5)

The solution of amide 4 (2.00 g, 9.9 mmol) in dry THF (180 ml) was added dropwise to an ice-cold solution of LiAlH_4_ (3.79 g, 100 mmol) in dry THF (180 ml) under argon atmosphere. After complete addition, the reaction mixture was refluxed for 7 hours. Then the mixture was cooled with an ice bath and decomposed according to Fieser workup. Precipitated salts were filtered of, washed several times with THF and the filtrate was evaporated. The residue was dissolved in dichloromethane (200 ml), washed with water (3 × 200 ml) and brine (150 ml), and the organic layer was dried with MgSO_4_. Purification of the crude product by column chromatography (CH_2_Cl_2_ : MeOH, 5 : 1) afforded *N*-methyltryptamine (5) as an off-white solid (717 mg, 42%). Mp = 62–64 °C (lit.^16^ 81–83 °C); ^1^H NMR (300 MHz, CDCl_3_) *δ*: 2.44 (s, 3H), 2.88–3.02 (m, 4H), 7.04 (d, 1H, *J* = 2.3 Hz), 7.09–7.15 (m, 1H), 7.17–7.23 (m, 1H), 7.35–7.39 (m, 1H), 7.62–7.66 (m, 1H), 8.06 (s br, 1H).

##### Ethyl 4-[*N*-[2-(1*H*-indol-3-yl)ethyl]-*N*-methyl]aminobutanoate (6)

To a mixture of *N*-methyltryptamine (5) (523 mg, 3.0 mmol) and NaI (450 mg, 3.0 mmol) in isopropyl alcohol (15 ml) was added DIPEA (620 mg, 835 μL, 4.8 mmol) and ethyl-4-bromobutyrate (878 mg, 645 μL, 4.5 mmol) and resulting solution was heated to 60 °C overnight. Solvent was removed on rotavap. The residue was dissolved in dichloromethane (75 ml) and washed with saturated NaHCO_3_ solution (75 ml), water (75 ml) and brine (75 ml). Organic phase was dried with MgSO_4_ and evaporated. Purification by column chromatography (CH_2_Cl_2_ : MeOH, 10 : 1) afforded the titled compound 5 as a light-brown viscous oil (570 mg, 65%). ^1^H NMR (300 MHz, CDCl_3_) *δ*: 1.25 (t, 3H, *J* = 7.3 Hz), 1.86 (qui, 2H, *J* = 7.2 Hz), 2.32–2.41 (m, 5H), 2.52 (t, 2H, *J* = 7.2 Hz), 2.72–2.81 (m, 2H), 2.92–3.01 (m, 2H), 4.13 (q, 2H, *J* = 7.3 Hz), 7.03 (d, 1H, *J* = 2.3 Hz), 7.08–7.14 (m, 1H), 7.15–7.22 (m, 1H), 7.33–7.38 (m, 1H), 7.58–7.63 (m, 1H), 8.09 (s br, 1H); ^13^C NMR (75 MHz, CDCl_3_) *δ*: 14.20, 22.29, 22.89, 32.07, 41.92, 56.56, 58.05, 60.32, 111.11, 114.02, 118.69, 119.15, 121.56, 121.87, 127.38, 136.20, 173.55; HRMS (ESI): *m*/*z* [M + H]^+^ calculated for C_17_H_24_N_2_O_2_: 289.19105, found 289.19125.

##### 4-[*N*-[2-(1*H*-indol-3-yl)ethyl]-*N*-methyl]aminobutanoic acid hydrochloride (1a, hapten I)

To a mixture of ester 5 (433 mg, 1.5 mmol) in 40% aqueous ethanol (20 ml) was added NaOH (66 mg, 1.65 mmol) and the solution was stirred at r.t. overnight. Then the solution was acidified with 1 M aqueous HCl to pH = 1, evaporated and the residue was purified by reverse-phase flash chromatography (MeOH : H_2_O, gradient 5–100% MeOH) to obtain the titled compound 1a (hapten I) as a white foam (246 mg, 63%). ^1^H NMR (300 MHz, DMSO-d_6_) *δ*: 1.83–1,96 (m, 2H), 2.35 (t, 2H, *J* = 7.3 Hz), 2.80 (s, 3H), 3.06–3.20 (m, 4H), 3.21–3.31 (m, 2H), 6.97–7.04 (m, 1H), 7.06–7.13 (m, 1H), 7.25 (d, 1H, *J* = 2.3 Hz), 7.37 (d, 1H, *J* = 8.2 Hz), 7.63 (d, 1H, *J* = 7.6 Hz), 11.00 (s br, 1H); ^13^C NMR (75 MHz, DMSO-d_6_) *δ*: 19.0, 19.8, 30.8, 39.3, 54.1, 55.2, 109.2, 111.5, 118.3, 118.5, 121.2, 123.2, 126.7, 136.2, 173.6; HRMS (ESI): *m*/*z* [M + H]^+^ calculated for C_15_H_20_N_2_O_2_: 261.15975, found 261.15979.

#### 4.1.2 Haptens II–IV

##### Ethyl 2-[4-[(*tert*-butoxycarbonyl)amino]phenoxy]acetate (16)

Compound 16 was prepared according to literature^17^ in two steps from 4-aminophenol (17) (10.09 g, 100.0 mmol) as a white solid (11.29 g, 88% (2 steps)). Mp = 53–55 °C; ^1^H NMR (300 MHz, CDCl_3_) *δ*: 1.27 (t, 3H, *J* = 7.2 Hz), 1.48 (s, 9H), 4.26 (q, 2H, *J* = 7.2 Hz), 4.56 (s, 2H) 6.55 (s br, 1H), 6.78–6.85 (m, 2H), 7.26 (d, 2H, *J* = 8.8 Hz); ^13^C NMR (75 MHz, CDCl_3_) *δ*: 14.1, 28.3, 61.3, 65.9, 80.2, 115.1, 120.3, 132.4, 153.0, 153.6, 169.0; HRMS (ESI): *m*/*z* [M + Na]^+^ calculated for C_15_H_21_NO_5_: 318.13119, found 318.13149.

##### 2-(4-aminophenoxy)acetic acid (8b)

Suspension of 16 (5.91 g, 20.0 mmol) in 1 M hydrochloric acid (50 ml) was stirred at 60 °C overnight. After cooling to r.t., the solution was extracted with dichloromethane (50 ml) and phases separated. Aqueous phase was treated with solid Na_2_CO_3_ to adjust pH to 4–5 (according to pH paper). The resulting precipitate was filtered off and dried, yielding the titled compound 8b as an off-white solid (3.03 g, 91%). Mp = 200–215 °C (lit.^18^ 200–210 °C); ^1^H NMR (300 MHz, DMSO-d_6_) *δ*: 4.46 (s, 2H), 6.45–6.52 (m, 2H), 6.58–6.65 (m, 2H).

##### General procedure A: preparation of arylhydrazinium chlorides

A procedure from the literature^19^ was modified as follows: suspension of aniline acid 8a,b (1 eq.) in concentrated hydrochloric acid (3 ml mmol^−1^) was cooled to −5 °C and a solution of sodium nitrite (1.05 eq.) in water (0.5 ml mmol^−1^) was added dropwise. After complete addition the mixture was stirred for 1 hour at −5 °C and then it was added dropwise to a solution of SnCl_2_·2H_2_O (3 eq.) in concentrated hydrochloric acid (1 ml mmol^−1^) cooled to −20 °C. After complete addition, the mixture was stirred for additional 2.5 hours at −20 °C and then the suspension was filtered. Solids were washed with cold ethanol and diethyl ether and dried, yielding arylhydrazinium chlorides 7a,b.

##### 4-(carboxymethyl)phenylhydrazinium chloride (7a)

Was prepared according to general procedure A from aniline 8a (2.27 g, 15.0 mmol) as a white solid (2.86 g, 94%). Mp = 207–210 °C (lit.^20^ 228–229 °C); ^1^H NMR (300 MHz, DMSO-d_6_) *δ*: 3.48 (s, 2H), 6.92 (d, 2H, *J* = 8.5 Hz), 7.16 (d, 2H, *J* = 8.5 Hz), 8.20 (s br, 1H), 10.27 (s br, 3H); ^13^C NMR (75 MHz, DMSO-d_6_) *δ*: 39.9, 114.6, 128.1, 129.9, 144.3, 173.0; HRMS (ESI): *m*/*z* [M]^+^ calculated for C_8_H_11_N_2_O_2_: 167.08150, found 167.08124.

##### 4-(carboxymethoxy)phenylhydrazinium chloride (7b)

Was prepared according to general procedure from aniline 8b (836 mg, 5.0 mmol) as a white solid (937 mg, 86%). Mp = 135 °C (decomp.); ^1^H NMR (300 MHz, DMSO-d_6_) *δ*: 4.60 (s, 2H), 6.82–6.89 (m, 2H), 6.95–7.02 (m, 2H), 10.63 (s br, 3H); ^13^C NMR (75 MHz, DMSO-d_6_) *δ*: 64.9; 115.0, 116.9, 139.5, 153.1, 170.3; HRMS (ESI): *m*/*z* [M − NH_3_]^+^ calculated for C_8_H_8_NO_3_: 166.04987, found 166.04977.

##### 4-Chloro-1,1-dimethoxybutane (12)

DIBAL-H (94 ml, 1 M solution in hexane, 94.0 mmol) was added dropwise to a solution of ethyl-4-chlorobutyrate (11) (12.30 g, 81.7 mmol) in dry dichloromethane (125 ml) cooled to −78 °C. After addition, the reaction mixture was stirred for half hour at −78 °C and then poured into ice-cold 10% hydrochloric acid (160 ml). Resulting mixture was stirred at 0 °C for 1 hour, then the layers were separated and the aqueous one extracted with dichloromethane (2 × 100 ml). Combined organic layers were washed with brine (300 ml) and dried with MgSO_4_. After solvent removal, the residue was dissolved in methanol (35 ml) acidified with few drops of concentrated sulfuric acid. The solution was stirred for 18 hours, diluted with dichloromethane (125 ml), washed with 10% aqueous NaHCO_3_ (100 ml), water (100 ml) and brine (100 ml) and dried with MgSO4. Distillation under reduced pressure gave the titled acetal 12 as a colorless liquid (9.76 g, 78%). Bp = 53–55 °C (5 torr) (lit.^21^ 84–86 °C (25 torr)); ^1^H NMR (300 MHz, CDCl_3_) *δ*: 1.70–1.90 (m, 4H), 3.32 (s, 6H), 3.56 (t, 2H, *J* = 6.3 Hz), 4.39 (t, 1H, *J* = 5.4 Hz).

##### 4-(*N*,*N*-dimethylamino)-1,1-dimethoxybutane (9)

Compound 9 was prepared according to literature^13^ from acetal 11 (4.86 g, 31.8 mmol) as a colorless liquid (4.32 g, 84%) after distillation under reduced pressure. Bp = 59–60 °C (6 torr) (lit.^13^ 40 °C (1 torr)).

##### 4-(*N*,*N*-diisopropylamino)-4-oxobutanoic acid (14)

To a solution of succinic anhydride (13) (10.07 g, 100.0 mmol) in dichloromethane (300 ml) was added diisopropyl amine (21.8 ml, 300.0 mmol) and resulting solution was stirred for 20 hours. Then the mixture was concentrated and 1 M hydrochloric acid (250 ml) was added to the residue. Aqueous layer was extracted with dichloromethane (3 × 150 ml) and combined organic layers were dried with MgSO4. Solvent removal offered the titled compound 14 as a light brown viscous oil (16.50 g, 82%), which solidified upon standing in a fridge. ^1^H NMR (300 MHz, CDCl_3_) *δ*: 1.16 (d, 6H, *J* = 6.7 Hz), 1.29 (d, 6H, *J* = 7.0 Hz), 2.56–2.64 (m, 4H), 3.46 (m br, 1H), 3.95 (sept, 1H, *J* = 6.7 Hz), 11.02 (s br, 1H); ^13^C NMR (75 MHz, CDCl_3_) *δ*: 20.3, 20.6, 29.5, 29.8, 45.9, 48.4, 170.7, 176.7.

##### 4-(*N*,*N*-diisopropylamino)butan-1-ol (15)

Suspension of LiAlH_4_ (6.83 g, 180.0 mmol) in dry THF (300 ml) was cooled with ice bath and then the solution of amide 14 (8.05 g, 40.0 mmol) in dry THF (150 ml) was added dropwise. After complete addition, the reaction mixture was refluxed for 5 hours. Then it was cooled with an ice bath and decomposed according to Fieser workup. Solids were removed by filtration, washed with THF and the filtrate was evaporated. The residue was dissolved in dichloromethane (250 ml), washed with water (3 × 200 ml) and brine (150 ml) and dried with MgSO_4_. Distillation under reduced pressure gave the titled amino alcohol 15 as a colorless liquid (4.91 g, 71%). Bp= 65–68 °C (0.43 torr); ^1^H NMR (300 MHz, CDCl_3_) *δ*: 1.04 (d, 12H, *J* = 6.7 Hz), 1.58–1.71 (m, 4H), 2.43–2.52 (m, 2H), 3.10 (sept, 2H, *J* = 6.7 Hz), 3.48–3.58 (m, 2H), 6.06 (s br, 1H); ^13^C NMR (75 MHz, CDCl_3_) *δ*: 20.0, 27.6, 32.4, 44.9, 47.5, 62.7.

##### 4-(*N*,*N*-diisopropylamino)-1,1-dimethoxybutane (10)

Dimethyl sulfoxide (2.56 ml, 36.0 mmol) was added dropwise to a solution of oxalyl chloride (2.57 ml, 30.0 mmol) in dry dichloromethane (60 ml) cooled to −55 °C and the mixture was stirred for additional 10 minutes. Then the solution of alcohol 15 (2.60 g, 15 mmol) in dry dichloromethane (10 ml) was added and the mixture was stirred at −55 °C for additional 15 minutes. Then trimethylamine (8.9 ml, 60.0 mmol) was added and the mixture was allowed to warm to r.t. (1 hour). Reaction mixture was then poured into water (130 ml), phases were separated and the aqueous one extracted with dichloromethane (2 × 100 ml). Combined organic phases were dried with MgSO_4_ and evaporated. The residue was dissolved in methanol (50 ml), acidified with concentrated sulfuric acid (2.5 ml) and the mixture was stirred at r.t. for 18 hours. Then the mixture was diluted with dichloromethane (150 ml), cooled with ice bath and 20% NaOH solution was added (100 ml). Phases were separated, the aqueous one was diluted with water (50 ml) and extracted with dichloromethane (2 × 75 ml). Combined organic phases were washed with brine (200 ml) and dried with MgSO_4_. Distillation under reduced pressure gave the titled amino acetal 10 as colorless liquid (1.74 g, 54%). Bp = 51–55 °C (0.24 torr); ^1^H NMR (300 MHz, CDCl_3_) *δ*: 0.98 (d, 12H, *J* = 6.6 Hz), 1.36–1.51 (m, 2H), 1.53–1.63 (m, 2H), 2.39 (t, 2H, *J* = 7.3 Hz), 2.99 (sept, 2H, *J* = 6.5 Hz), 3.31 (s, 6H), 4.37 (t, 1H, *J* = 5.9 Hz); ^13^C NMR (75 MHz, CDCl_3_) *δ*: 20.7, 26.1, 30.3, 44.7, 48.2, 52.6, 104.7.

##### General procedure B: preparation of haptens II–IV

A procedure from the literature^13^ was modified as follows: to a 4% sulfuric acid, which was first heated to 50 °C and bubbled with argon, arylhydrazinium chloride 7a,b (1 eq.) and then amino acetal 9 or 10 (1.2 eq.) were added and the resulting mixture was heated to 80 °C for 3.5 hours. After cooling to r.t., the mixture was neutralized with concentrated ammonia solution. Water was removed under reduced pressure and the residue was treated with ethanol (10 ml mmol^−1^) and filtered to remove most of the inorganic salts. Filtrate was evaporated, 1 M hydrochloric acid (10 ml mmol^−1^) was added and resulting solution was evaporated again. Purification of crude product by reverse-phase flash chromatography (water/methanol, gradient 5–100% of methanol) gave haptens 1b–d (haptens II–IV).

##### 2-[3-[2-(*N*,*N*-dimethylamino)ethyl]-1*H*-indol-5-yl]acetic acid hydrochloride (1b, hapten II)

Was prepared according to general procedure B from arylhydrazinium chloride 7a (608 mg, 3.0 mmol) and acetal 9 (580 mg, 3.6 mmol) as a colorless glassy solid (475 mg, 56%). ^1^H NMR (300 MHz, CD_3_OD) *δ*: 2.87 (s, 6H), 3.11–3.21 (m, 2H), 3.30–3.39 (m, 2H), 3.66 (s, 2H), 7.07 (dd, 1H, *J*_1_ = 8.5 Hz, *J*_2_ = 1.5 Hz), 7.15 (s, 1H), 7.30 (d, 1H, *J* = 8.5 Hz), 7.51 (s, 1H); ^13^C NMR (75 MHz, D_2_O) *δ*: 20.0, 41.4, 42.6, 57.5, 108.3, 112.2, 118.7, 123.6, 124.6, 125.6, 126.7, 135.5, 178.3; HRMS (ESI): *m*/*z* [M]^+^ calculated for C_14_H_18_N_2_O_2_: 247.14410, found 247.14413.

##### 2-[3-[2-(*N*,*N*-dimethylamino)ethyl]-1*H*-indol-5-yloxy]acetic acid hydrochloride (1c, hapten III)

Was prepared according to general procedure B from arylhydrazinium chloride 7b (328 mg, 1.5 mmol) and acetal 9 (290 mg, 1.8 mmol) as a colorless glassy solid (238 mg, 53%). ^1^H NMR (300 MHz, D_2_O) *δ*: 2.72 (s, 6H), 2.90–3.00 (m, 2H), 3.08–3.18 (m, 2H), 4.26 (s, 2H), 6.73 (dd, 1H, *J*_1_ = 8.8 Hz, *J*_2_ = 2.1 Hz), 6.99 (d, 1H, *J* = 2.1 Hz), 7.10 (s, 1H), 7.21 (d, 1H, *J* = 8.8 Hz); ^13^C NMR (75 MHz, D_2_O) *δ*: 20.1, 42.7, 57.4, 67.7, 100.9, 108.2, 112.3, 112.9, 124.8, 126.6, 131.7, 152.0, 177.2; HRMS (ESI): *m*/*z* [M]^+^ calculated for C_14_H_18_N_2_O_3_: 263.13902, found 263.13912.

##### 2-[3-[2-(*N*,*N*-diisopropylamino)ethyl]-1*H*-indol-5-yl]acetic acid hydrochloride (1d, hapten IV)

Was prepared according to general procedure B from arylhydrazinium chloride 7a (203 mg, 1.0 mmol) and acetal 10 (262 mg, 1.2 mmol) as a white solid (207 mg, 61%). ^1^H NMR (300 MHz, CD_3_OD) *δ*: 1.26 (d, 12H, *J* = 6.5 Hz), 2.83–3.04 (m, 4H), 3.48 (sept, 2H, *J* = 6.5 Hz), 3.57 (s, 2H), 6.93 (s, 1H), 7.11 (dd, 1H, *J*_1_ = 8.5 Hz, *J*_2_ = 1.5 Hz), 7.24 (d, 1H, *J* = 8.5 Hz), 7.38 (s, 1H); ^13^C NMR (75 MHz, CD_3_OD) *δ*: 18.1, 24.9, 46.6, 49.2, 55.9, 110.2, 112.3, 119.1, 124.4, 124.5, 128.1, 129.8, 136.7, 180.9.

## 5. Conclusion

We successfully used haptens with novel structures to produce polyclonal antibodies against various tryptamines. The constructed ELISAs have low detection limits. Some of the antibodies show good reactivity not only with the target analytes, but also with psilocin and 5-MeO-DiPT. Although the antibodies have not yet been characterized in complex matrices, they appear to be suitable for the development of immunochemical assay kits. In our next work, we will focus on the establishment of an ELISA for the detection of tryptamines in human body fluids. We believe that the outcome of our work could lead to LFIA kits designed for the on-site testing of NPS users.

## Conflicts of interest

None.

## Supplementary Material

RA-008-C8RA02528B-s001
