# Supplementary material for: Use of novel haptens in the production of antibodies for the detection of tryptamines
Source: RSC Adv. 2018 May 1;8(29):16243–50. doi: 10.1039/c8ra02528b (PMC9080274; doi:10.1039/c8ra02528b)

Electronic Supplementary Material (ESI) for RSC Advances.
This journal is © The Royal Society of Chemistry 2018

Supplementary information

Use of novel haptens in the production of antibodies for the detection of tryptamines

Michal Maryška[a, d], Lucie Fojtíková[a, c], Radek Jurok[b, d], Barbora Holubová[c], Oldřich Lapčík[a] and Martin Kuchař[a, d,*]

[a] *Department of Chemistry of Natural Compounds, University of Chemistry and Technology, Technická 5, 166 28 Praha 6 - Dejvice, Czech Republic*

[b] *Department of Organic Chemistry, University of Chemistry and Technology, Technická 5, 166 28 Praha 6 - Dejvice, Czech Republic*

[c] *Department of Biochemistry and Microbiology, University of Chemistry and Technology, Technická 3, 166 28 Praha 6 - Dejvice, Czech Republic*

[d] *Forensic Laboratory of Biologically Active Substances, University of Chemistry and Technology, Technická 3, 166 28 Praha 6 - Dejvice, Czech Republic*

∗ Corresponding author. Tel.: +420-220-444-432; fax: +420-220-444-422; e-mail: martin.kuchar@vscht.cz

Table of contents

^{1}H and ^{13}C NMR spectra of prepared compounds .... **p2 - p31**

^{1}H NMR of 2-(*1H*-indol-3-yl)-2-oxoacetyl chloride (**3**) (300 MHz, DMSO-d_6)

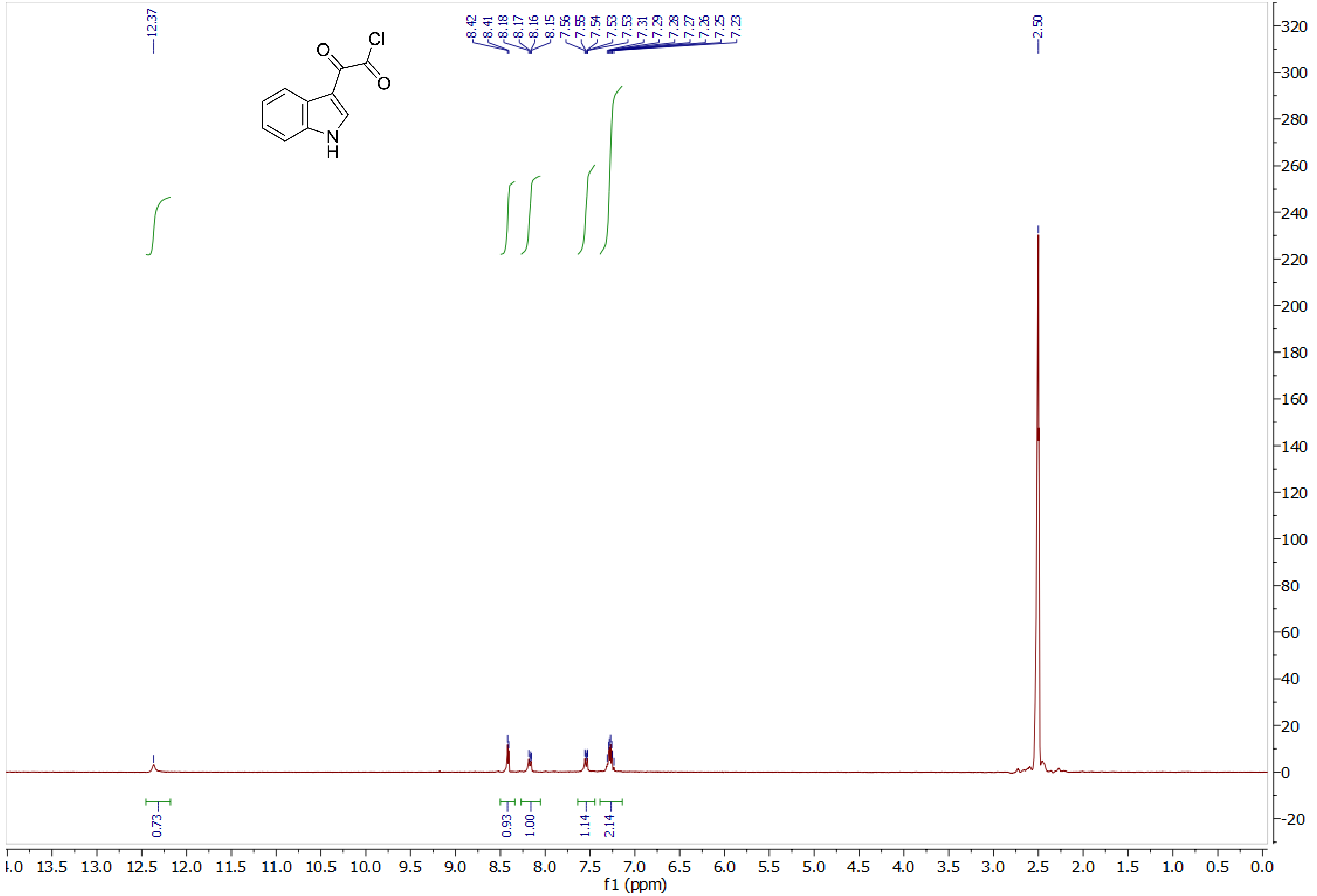

^{1}H NMR of 2-(*1H*-indol-3-yl)-*N*-methyl-2-oxoacetamide (**4**) (300 MHz, DMSO-d_6)

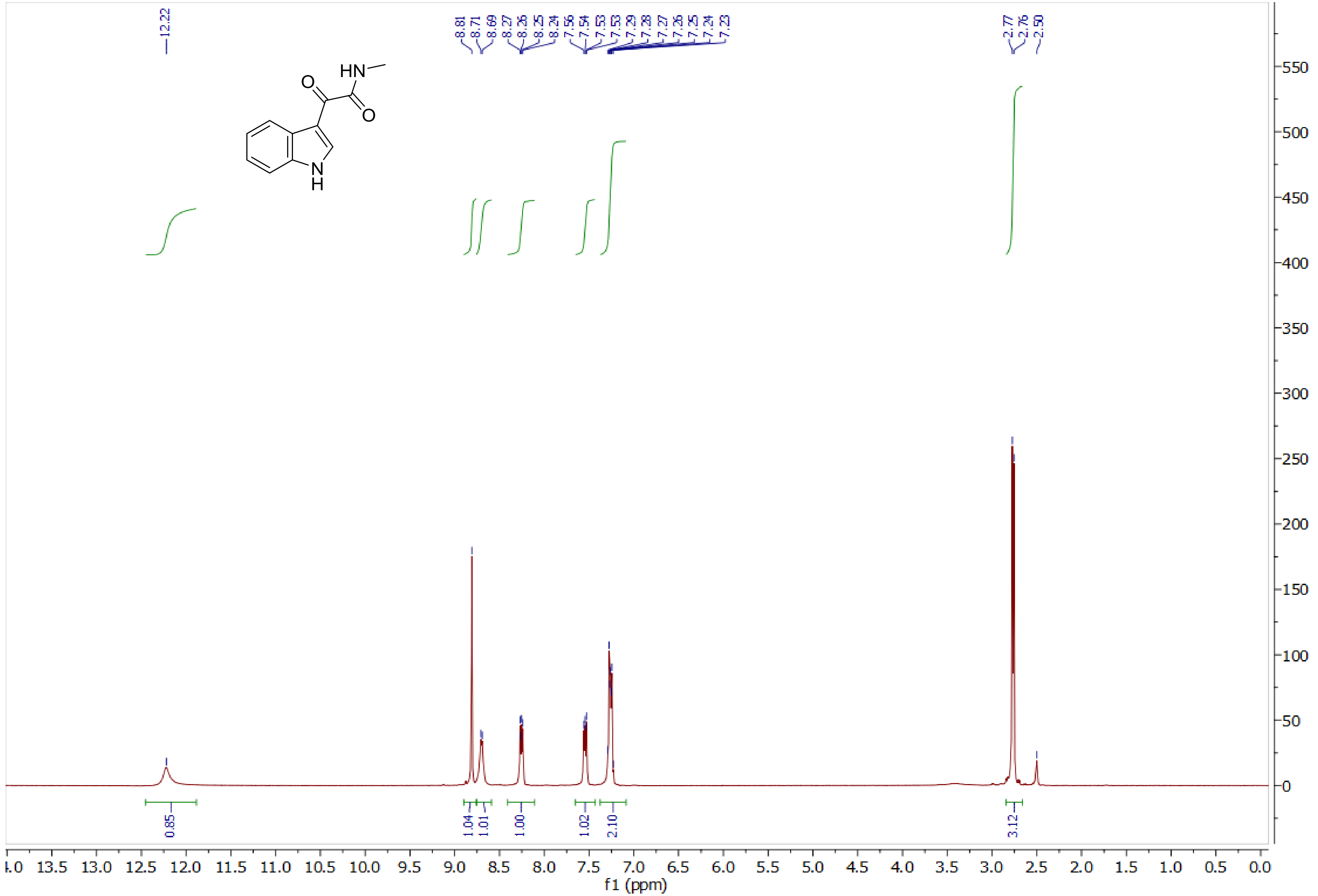

^{13}C NMR of 2-(*1H*-indol-3-yl)-*N*-methyl-2-oxoacetamide (**4**) (75 MHz, DMSO-d_6)

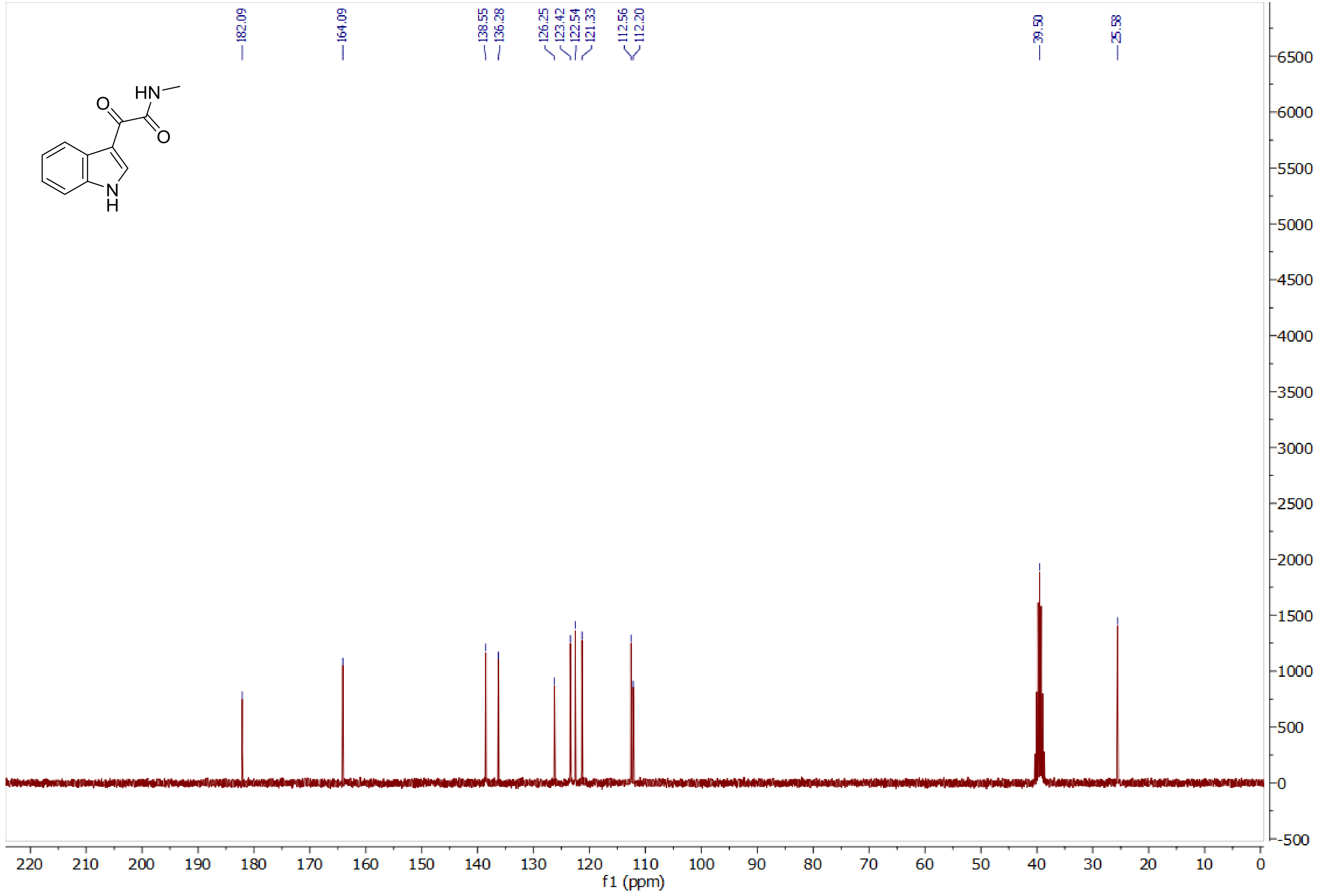

^{1}H NMR of *N*-methyltryptamine (**5**) (300 MHz, $CDCl_3$)

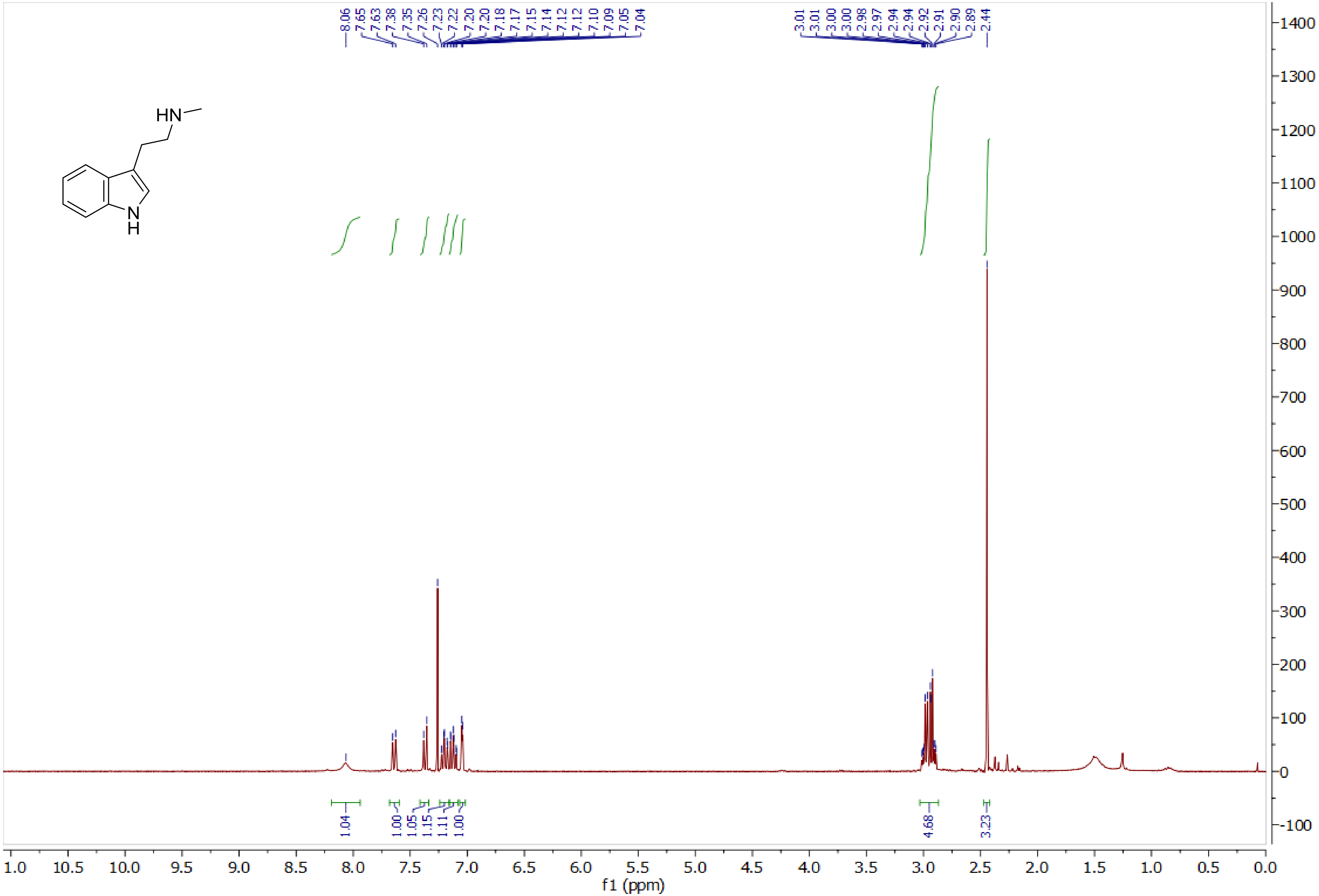

^{1}H NMR of ethyl 4-[*N*-[2-(*1H*-indol-3-yl)ethyl]-*N*-methyl]aminobutanoate (**6**) (300 MHz, $CDCl_3$)

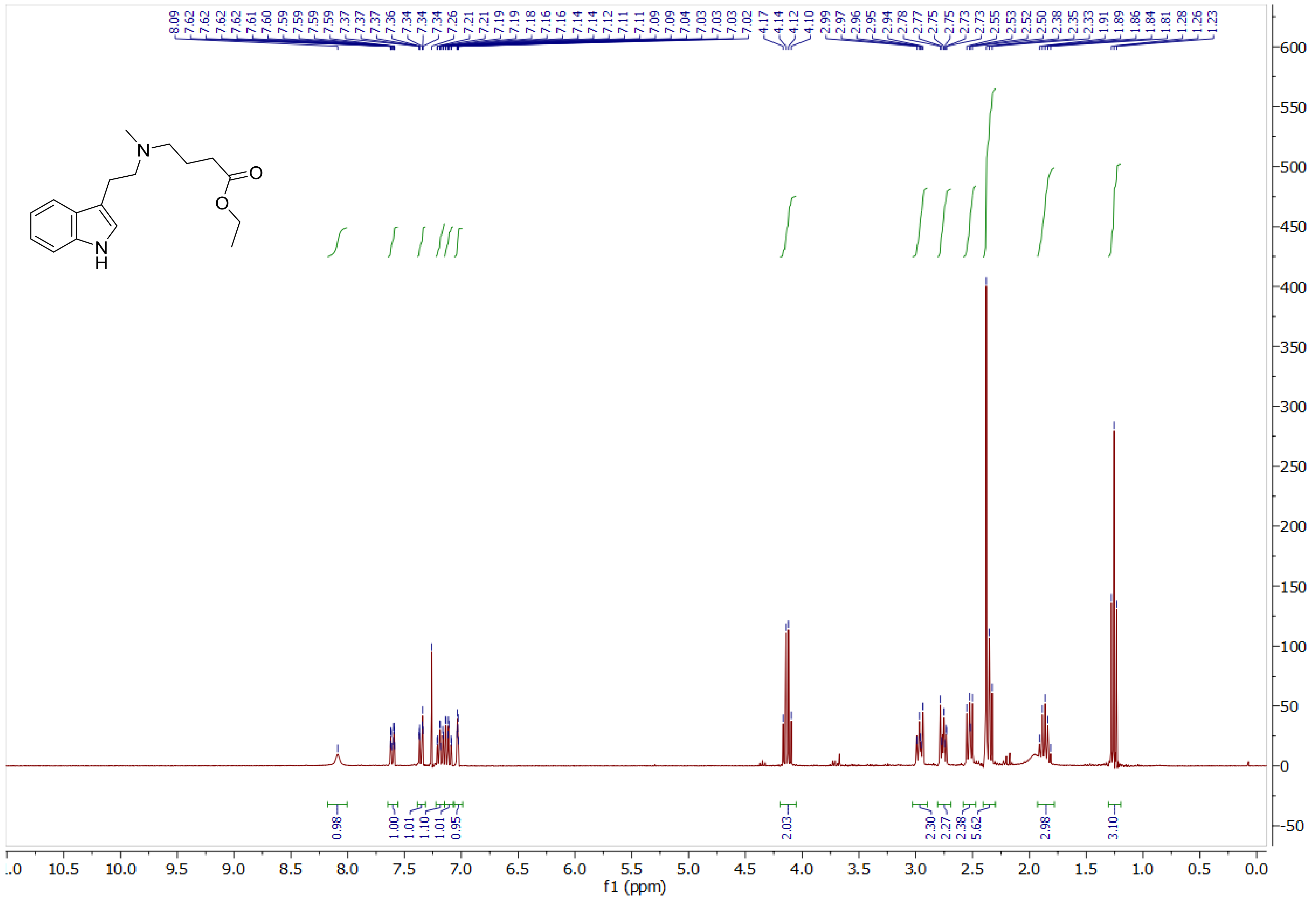

^{13}C NMR of ethyl 4-[*N*-[2-(*1H*-indol-3-yl)ethyl]-*N*-methyl]aminobutanoate (**6**) (75 MHz, $CDCl_3$)

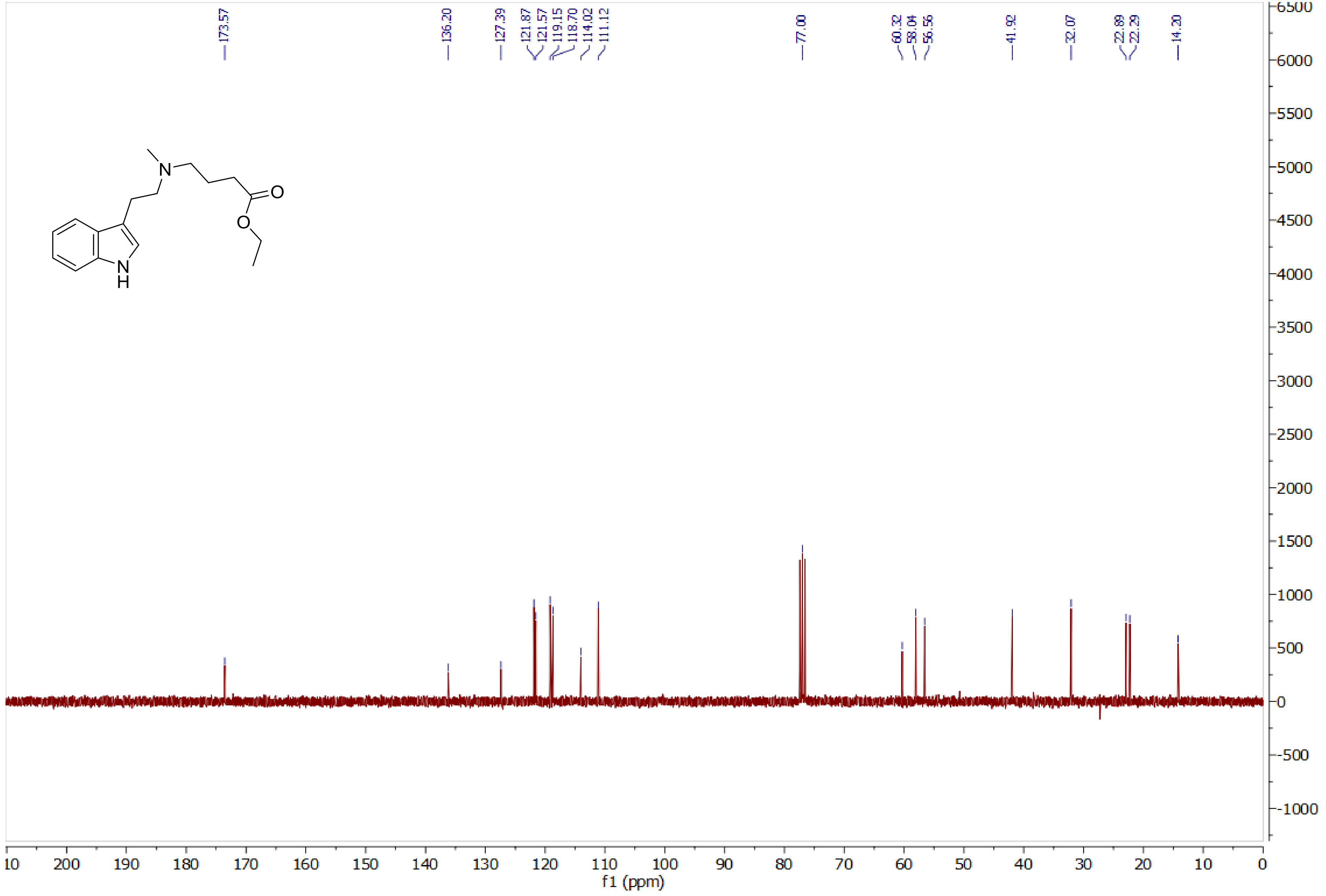

^{1}H NMR of ethyl 2-[4-[(*tert*-butoxycarbonyl)amino]phenoxy]acetate (**16**) (300 MHz, $CDCl_3$)

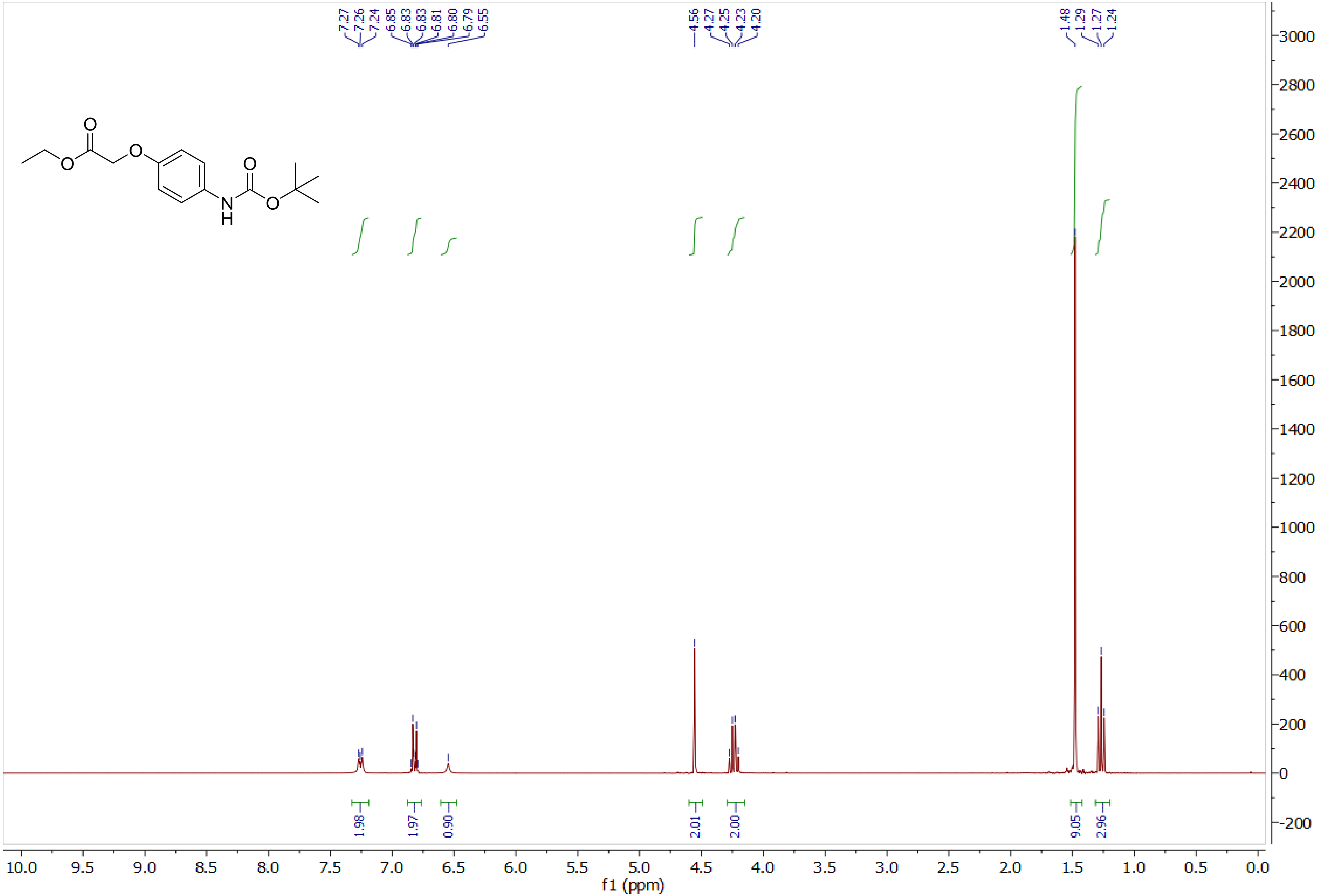

^{13}C NMR of ethyl 2-[4-[(*tert*-butoxycarbonyl)amino]phenoxy]acetate (**16**) (75 MHz, $CDCl_3$)

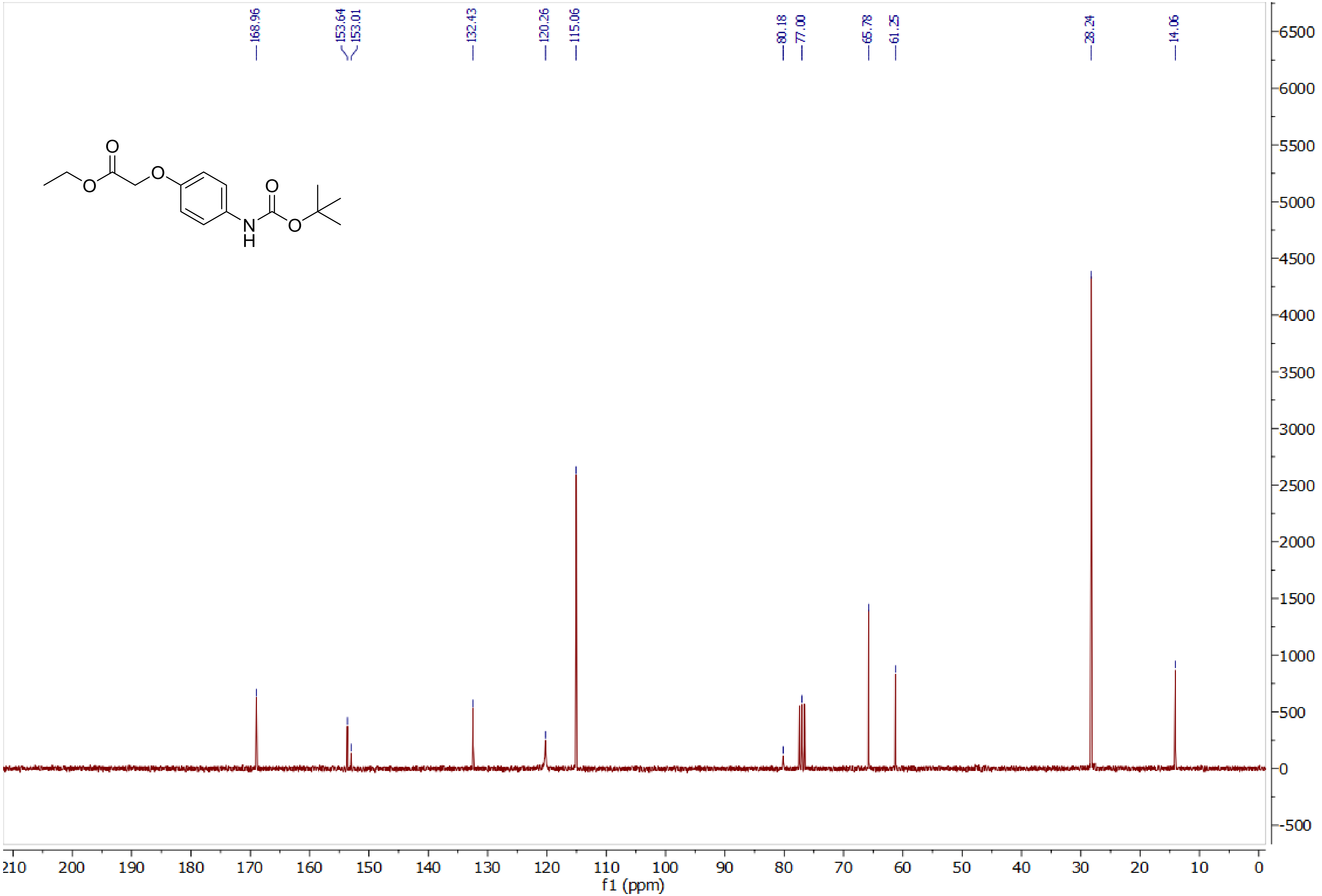

^{1}H NMR of 2-(4-aminophenoxy)acetic acid (**8b**) (300 MHz, DMSO-d_6)

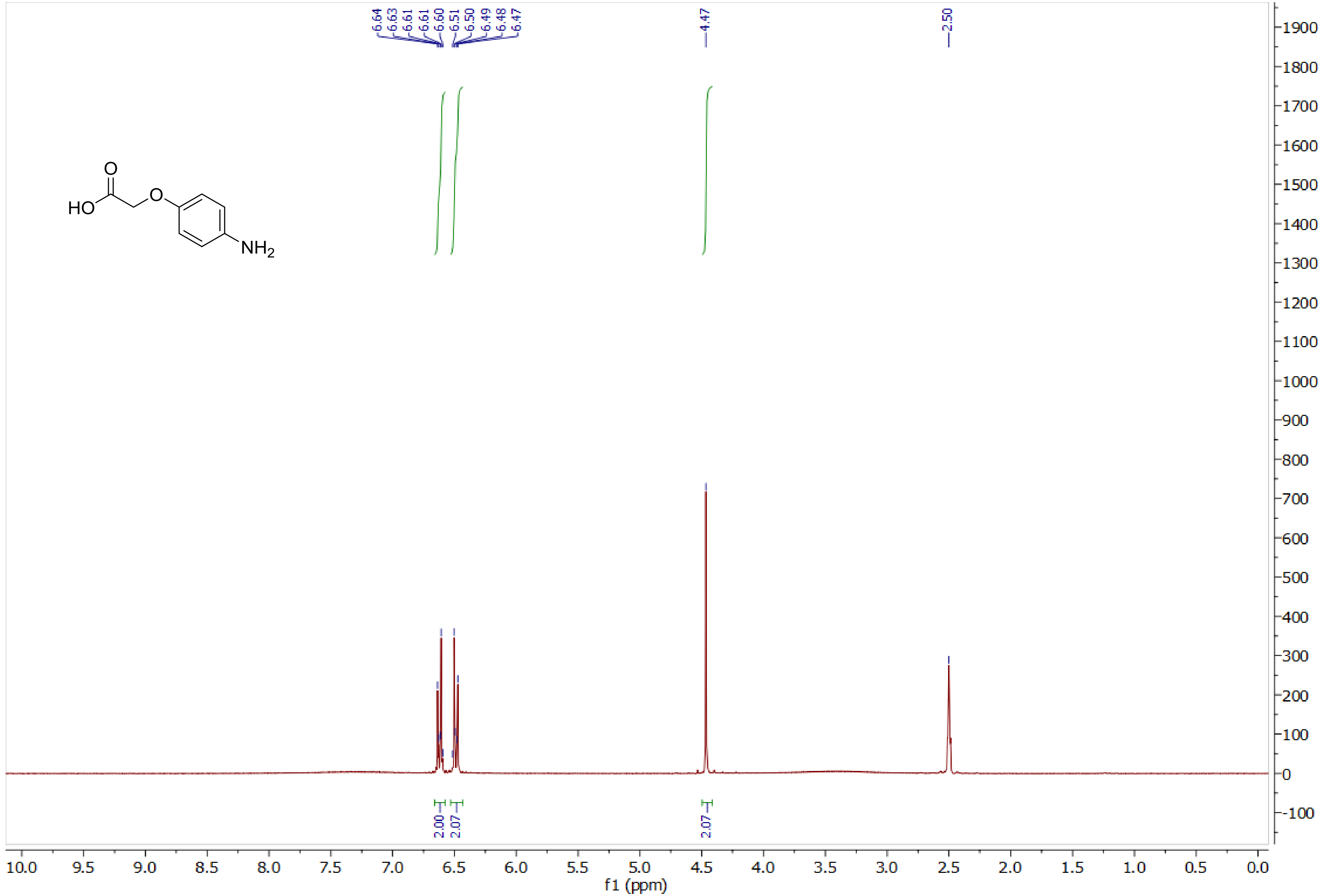

^{13}C NMR of 2-(4-aminophenoxy)acetic acid (**8b**) (75 MHz, DMSO-d$_6$)

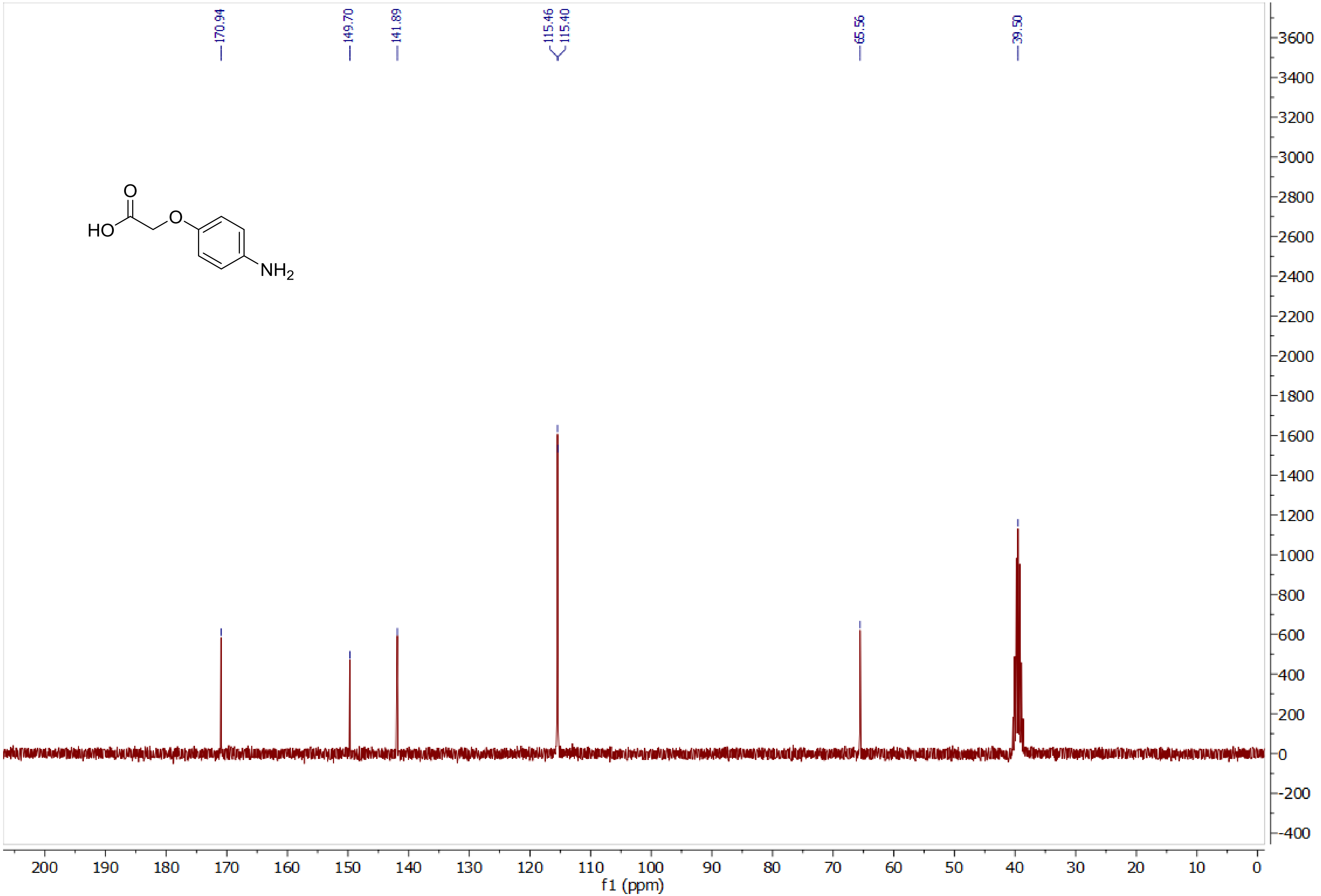

^{1}H NMR of 4-(carboxymethyl)phenylhydrazinium chloride (**7a**) (300 MHz, DMSO-d_6)

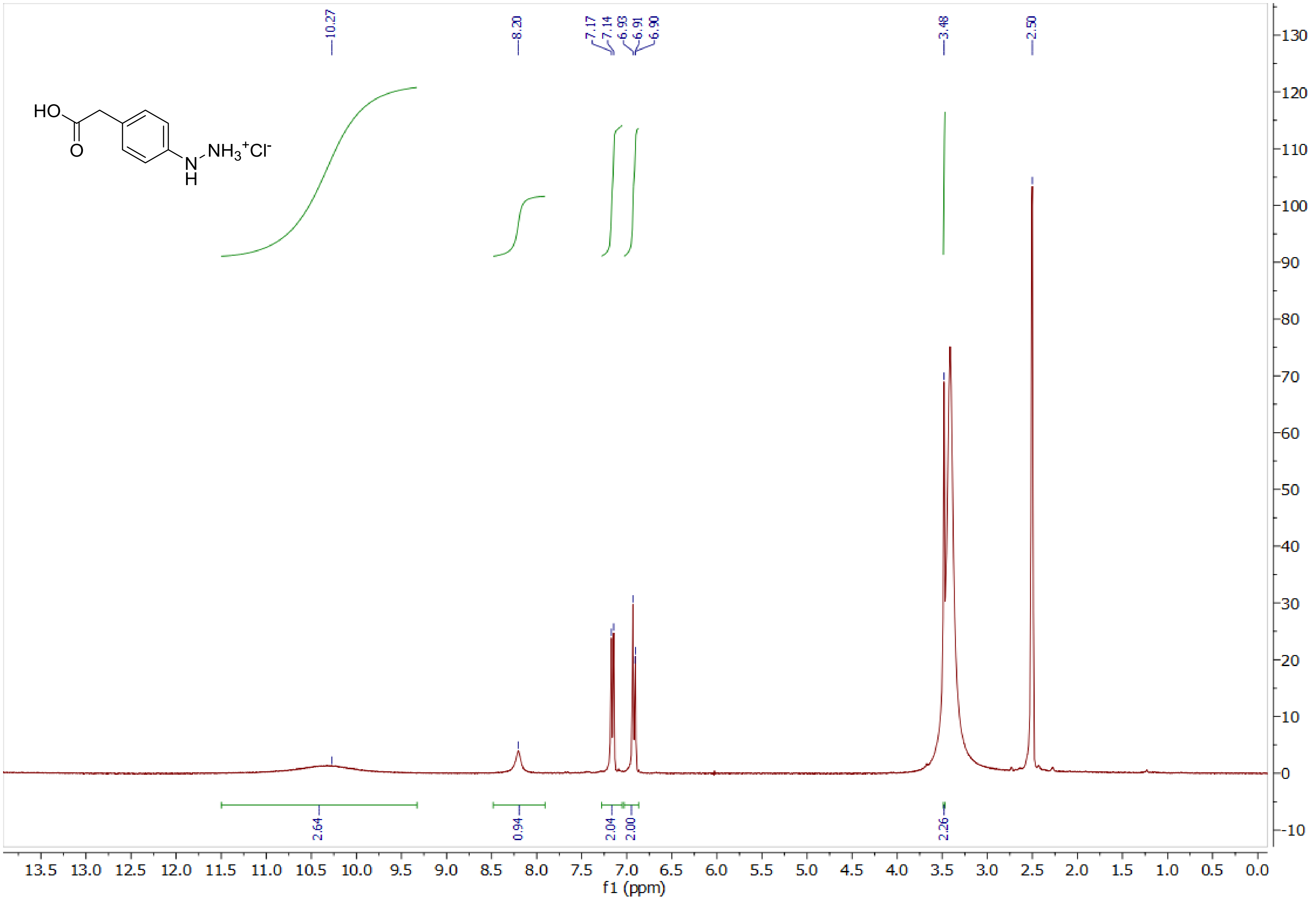

^{13}C NMR of 4-(carboxymethyl)phenylhydrazinium chloride (**7a**) (75 MHz, DMSO-d_6)

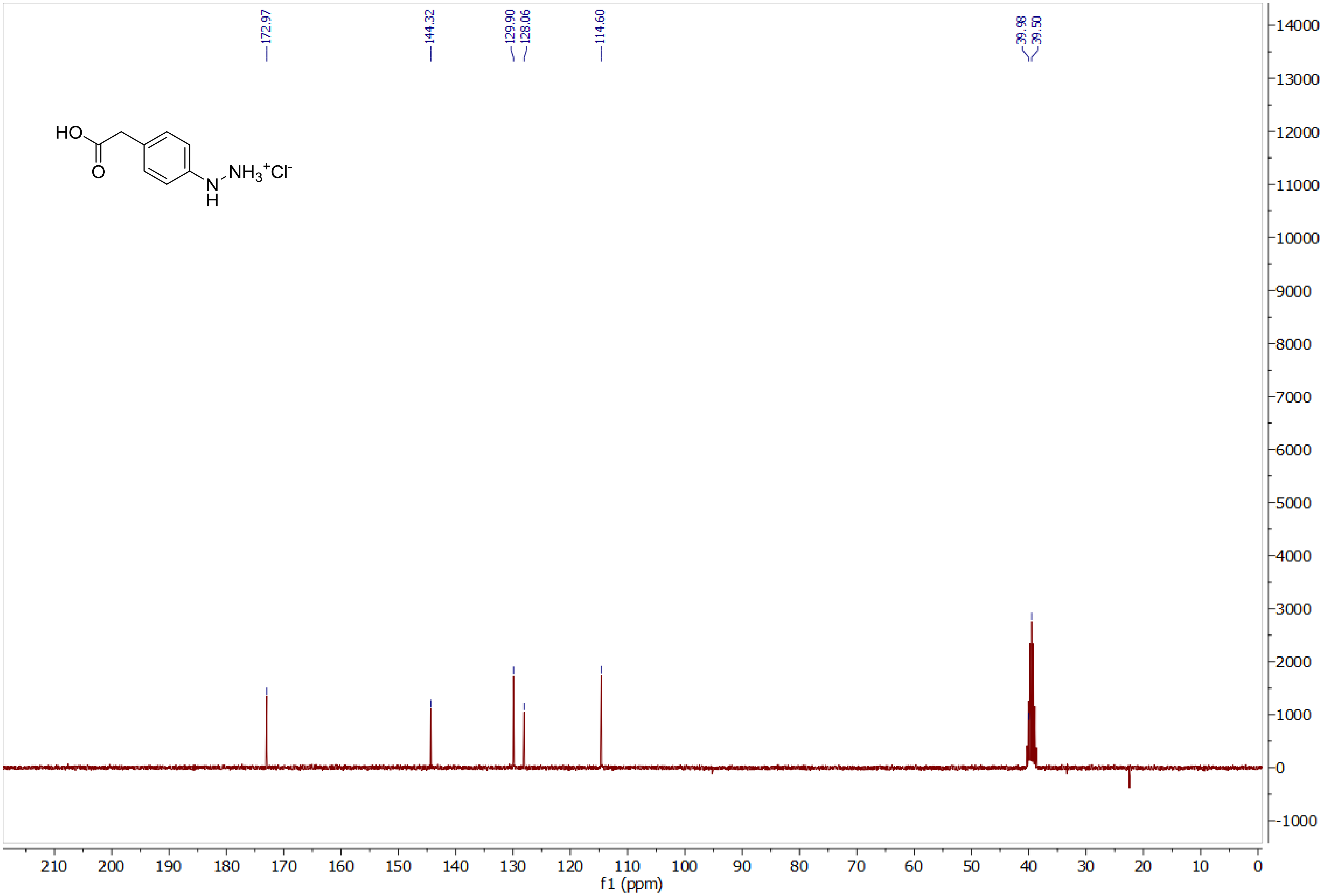

^{1}H NMR of 4-(carboxymethoxy)phenylhydrazinium chloride (**7b**) (300 MHz, DMSO-d_6)

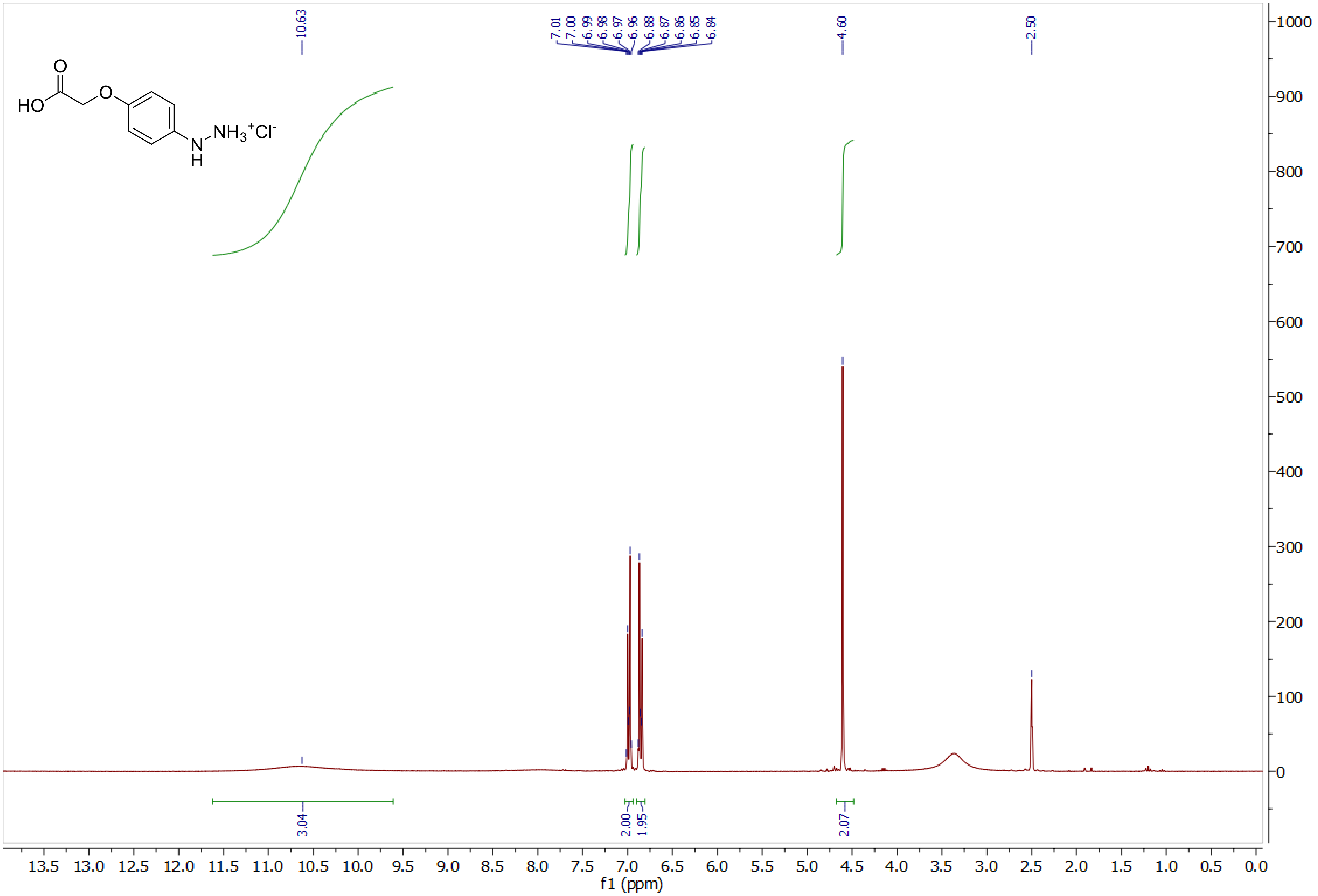

^{13}C NMR of 4-(carboxymethoxy)phenylhydrazinium chloride (**7b**) (75 MHz, DMSO-d_6)

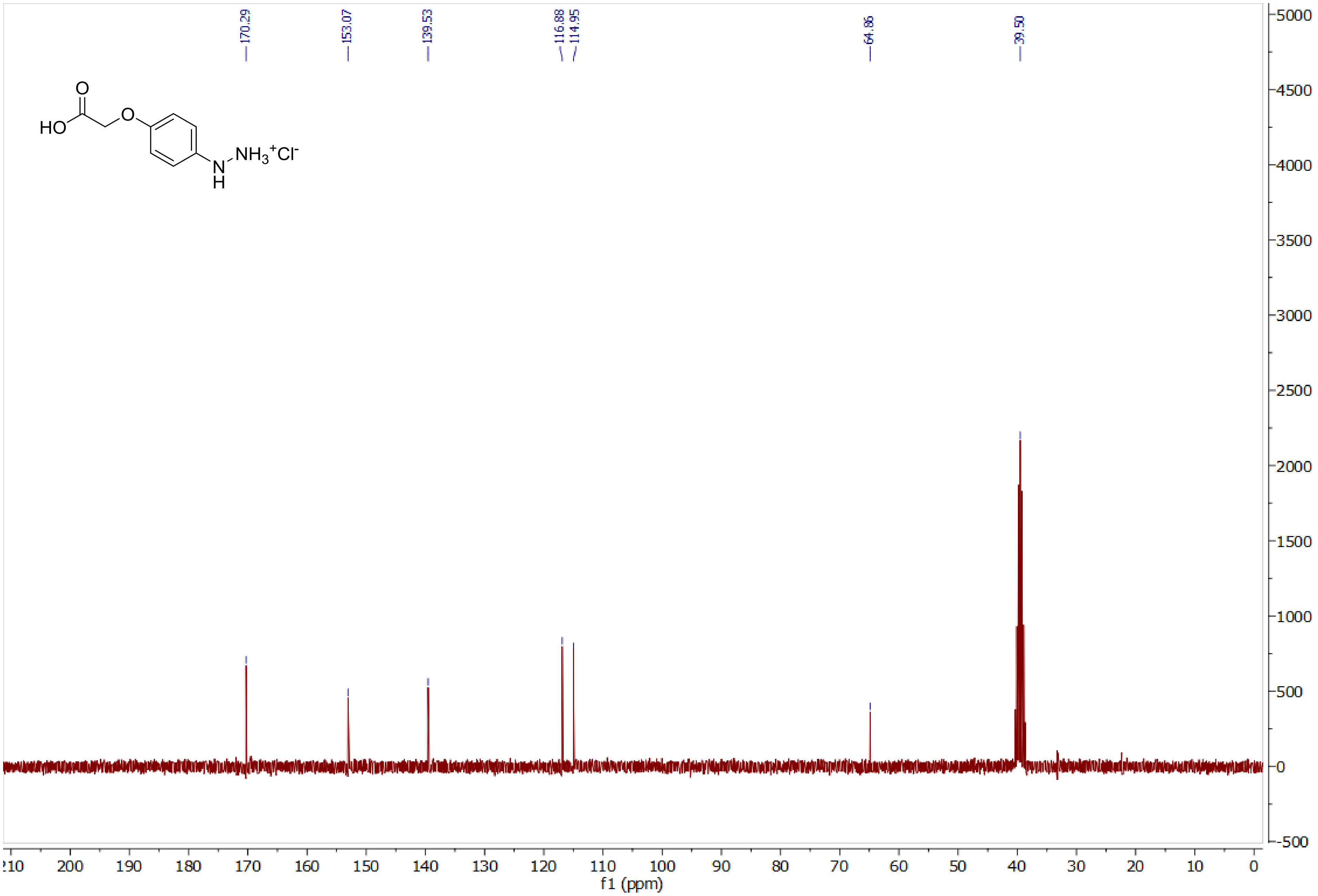

^{1}H NMR of 4-chloro-1,1-dimethoxybutane (**12**) (300 MHz, $CDCl_3$)

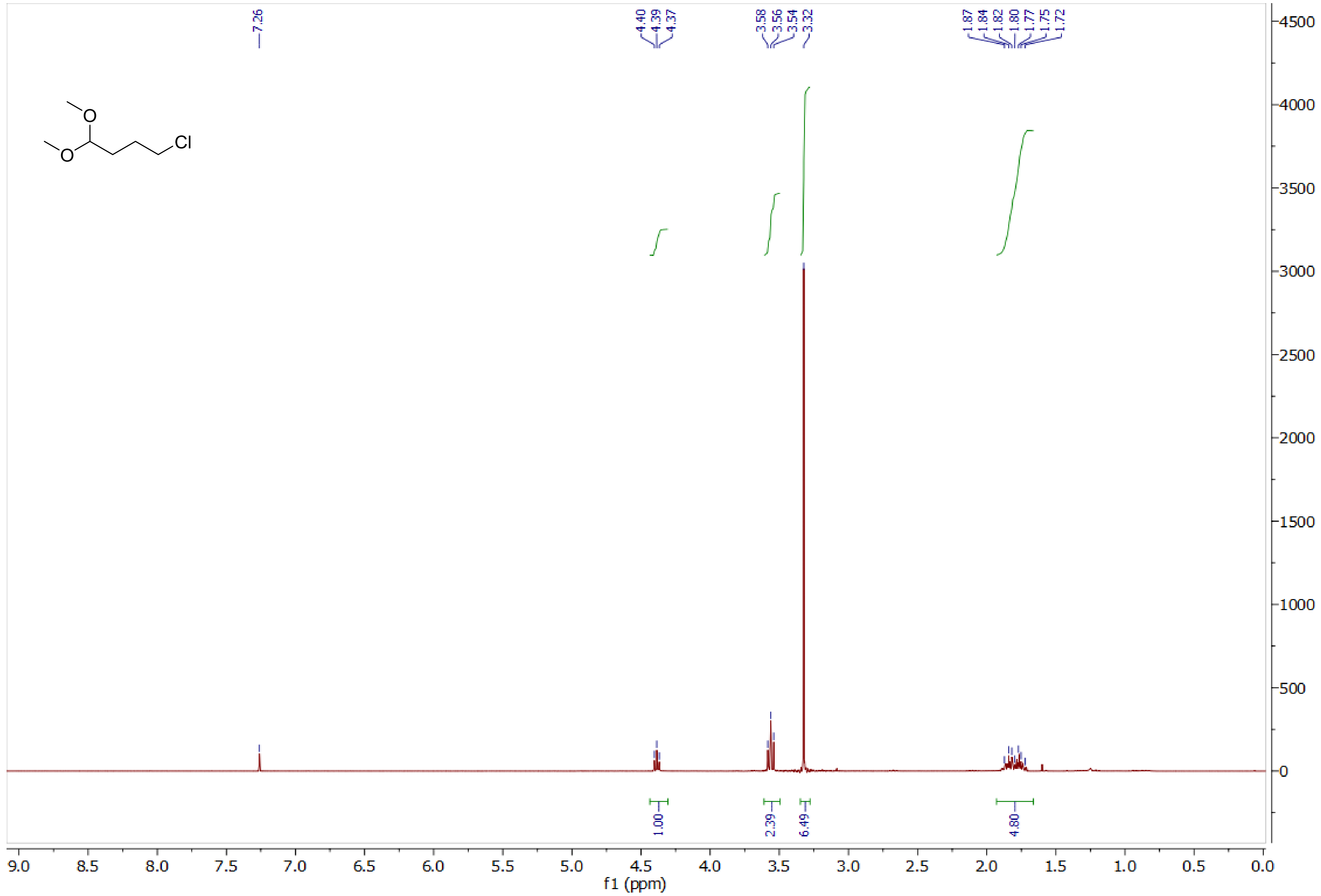

^{1}H NMR of 4-(*N*,*N*-dimethylamino)-1,1-dimethoxybutane (**9**) (300 MHz, $CDCl_3$)

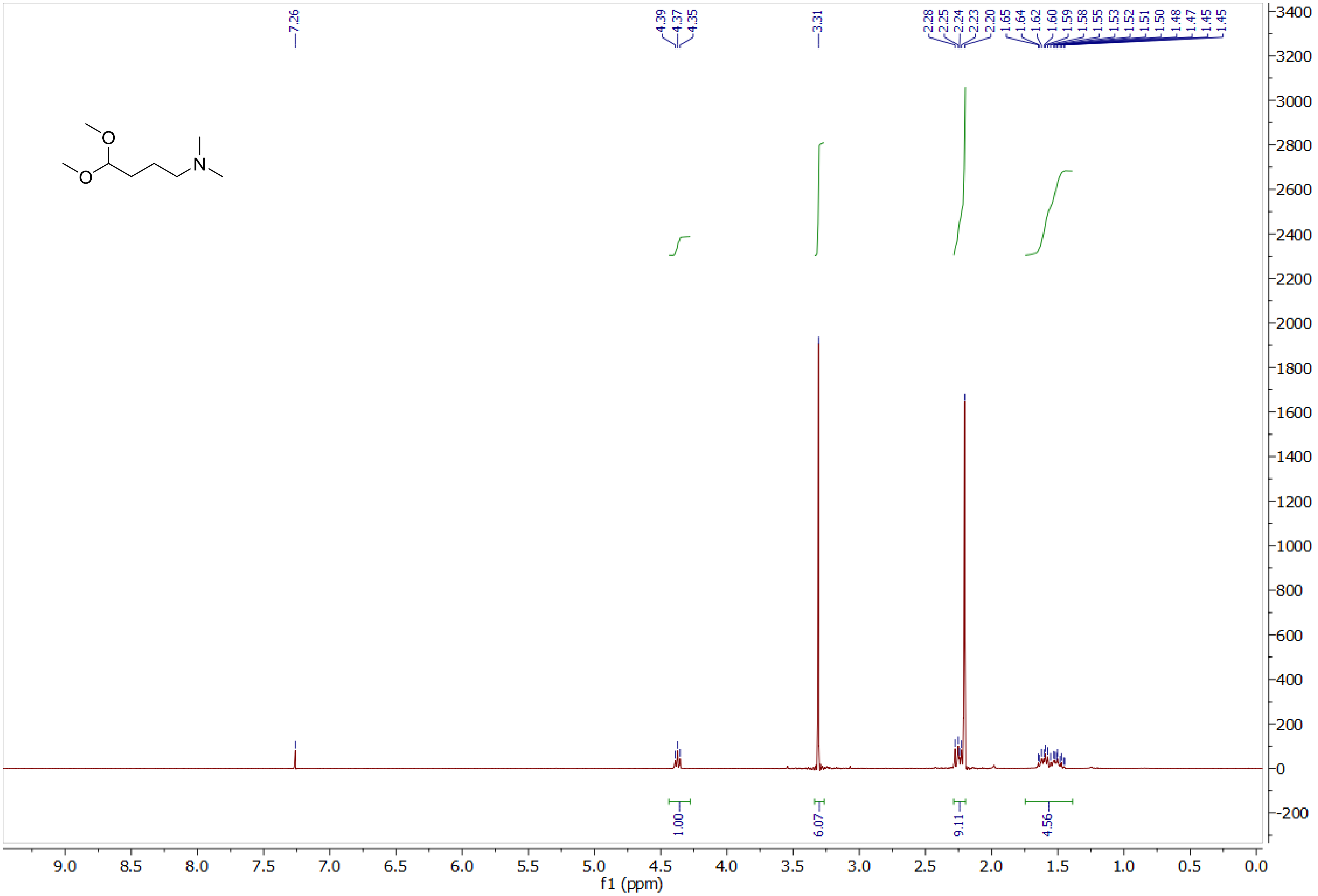

^{1}H NMR of 4-(*N*,*N*-diisopropylamino)-4-oxobutanoic acid (**14**) (300 MHz, $CDCl_3$)

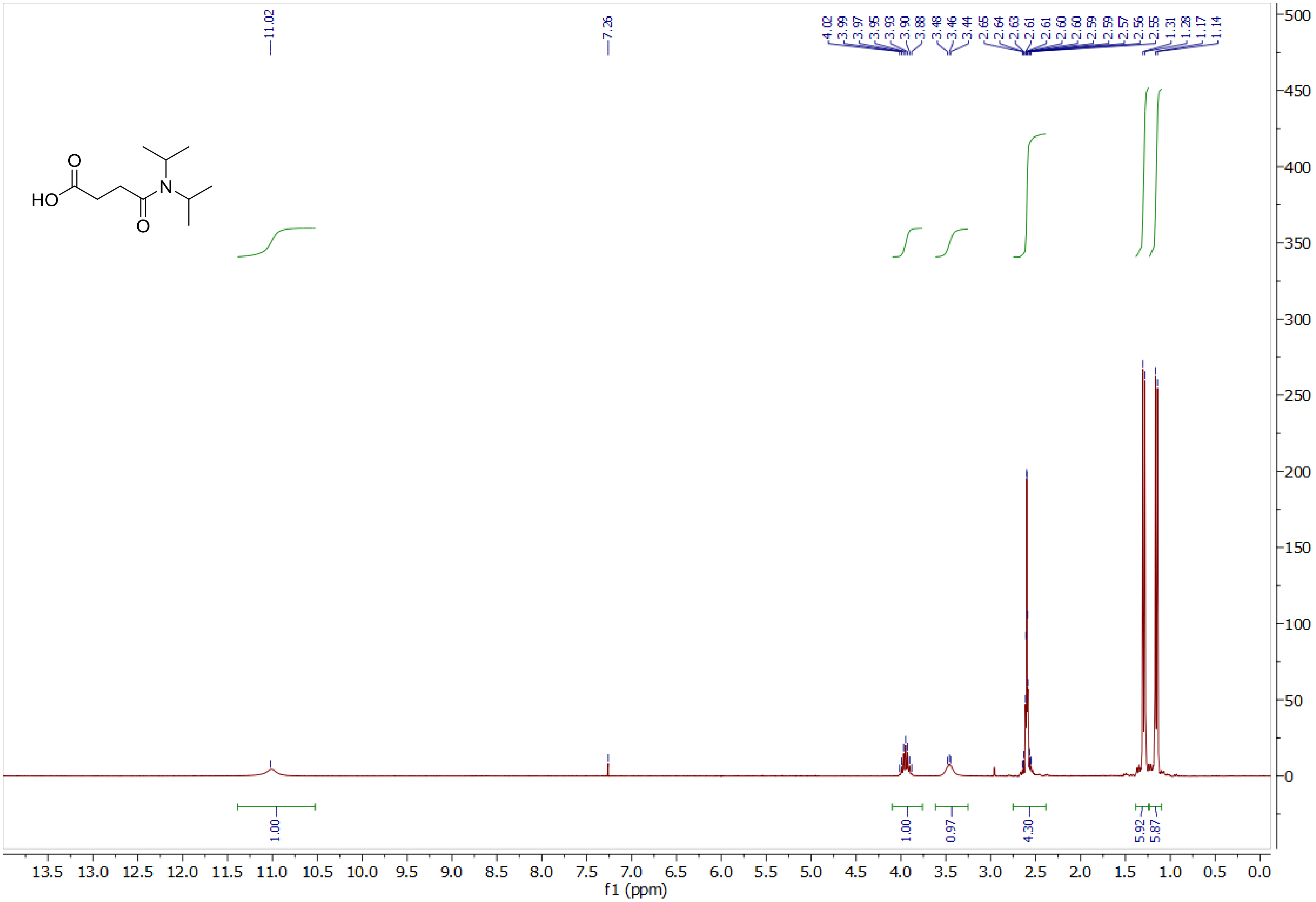

^{13}C NMR of 4-(*N*,*N*-diisopropylamino)-4-oxobutanoic acid (**14**) (75 MHz, $CDCl_3$)

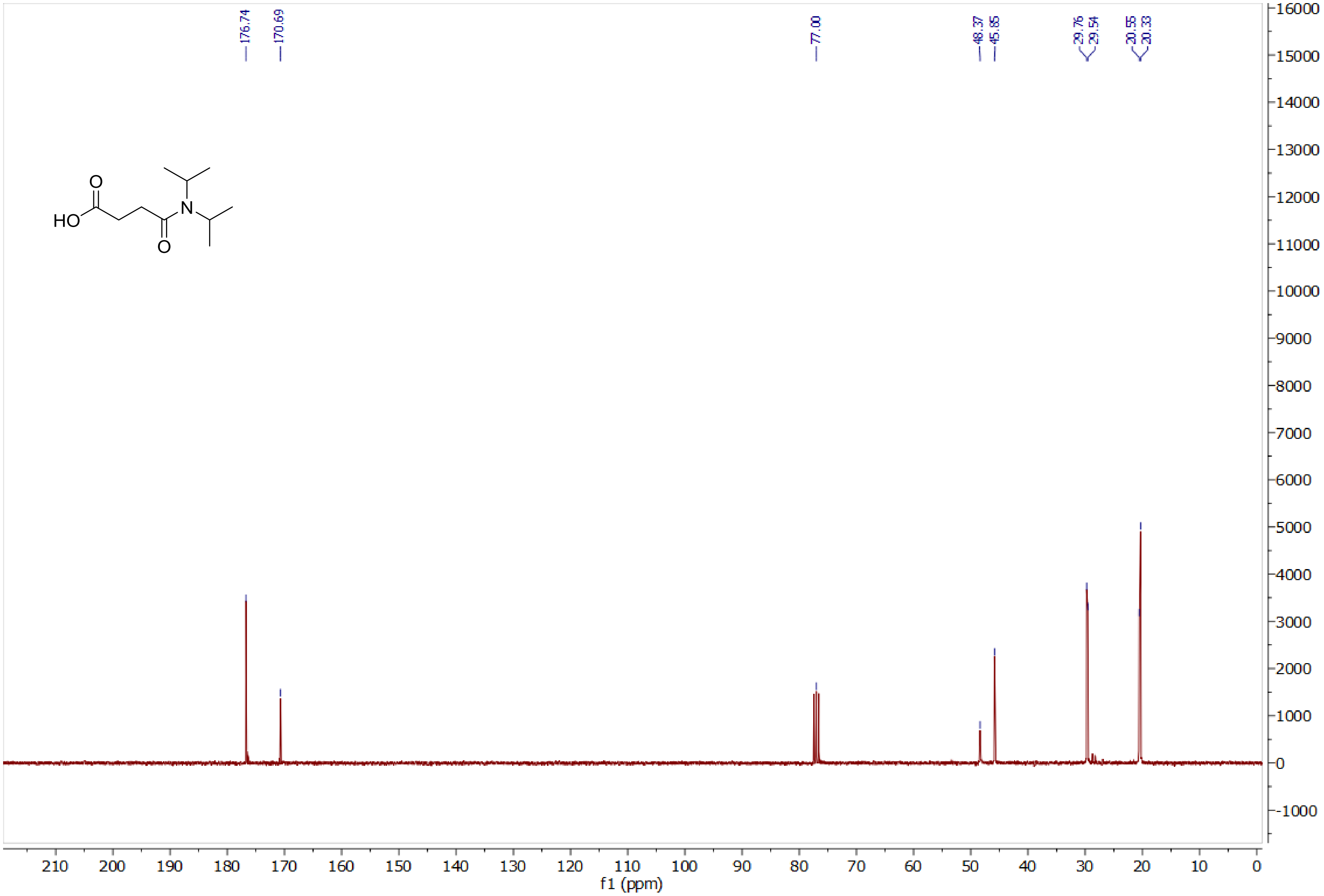

^{1}H NMR of 4-(*N*,*N*-diisopropylamino)butan-1-ol (**15**) (300 MHz, $CDCl_3$)

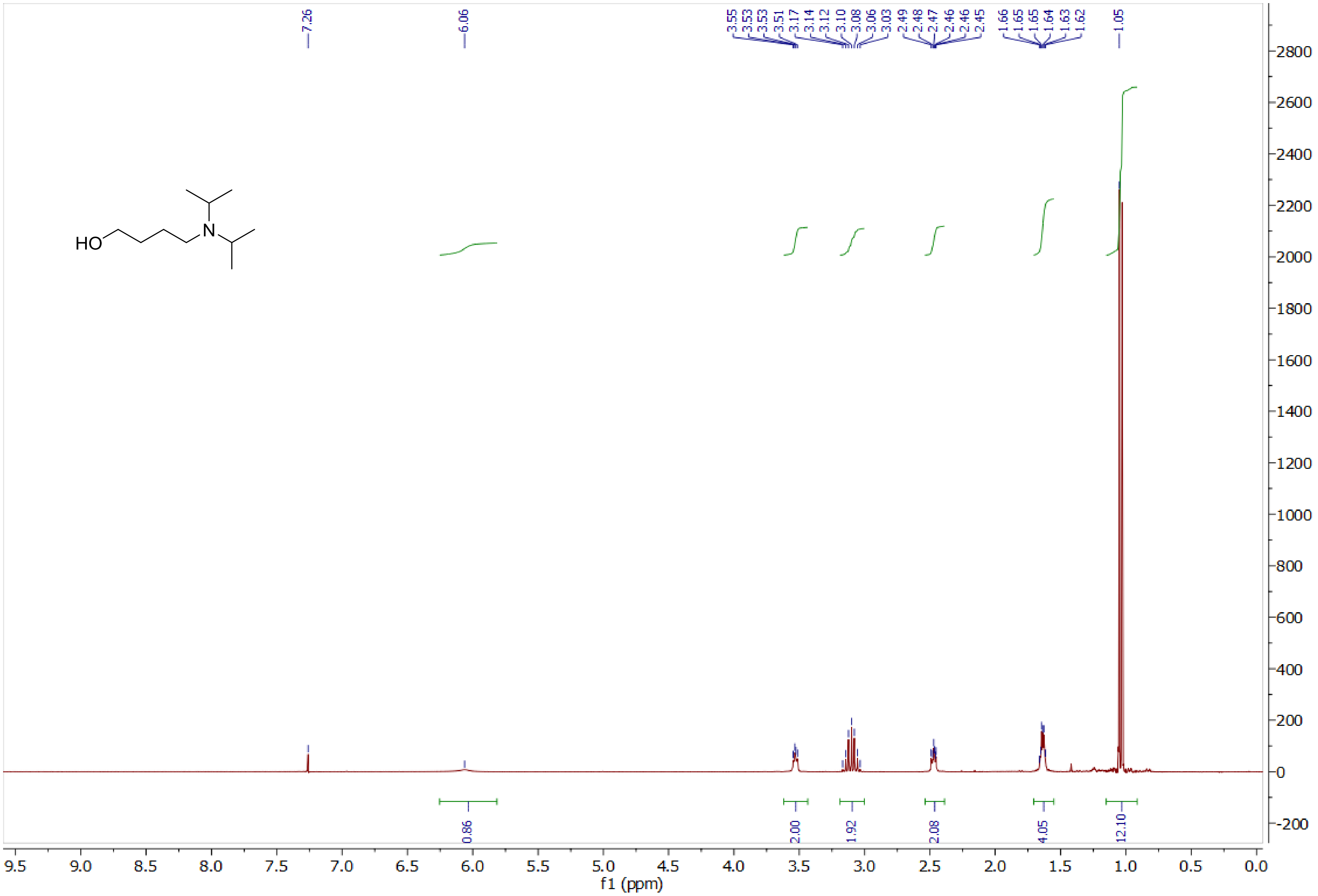

^{13}C NMR of 4-(*N*,*N*-diisopropylamino)butan-1-ol (**15**) (75 MHz, $CDCl_3$)

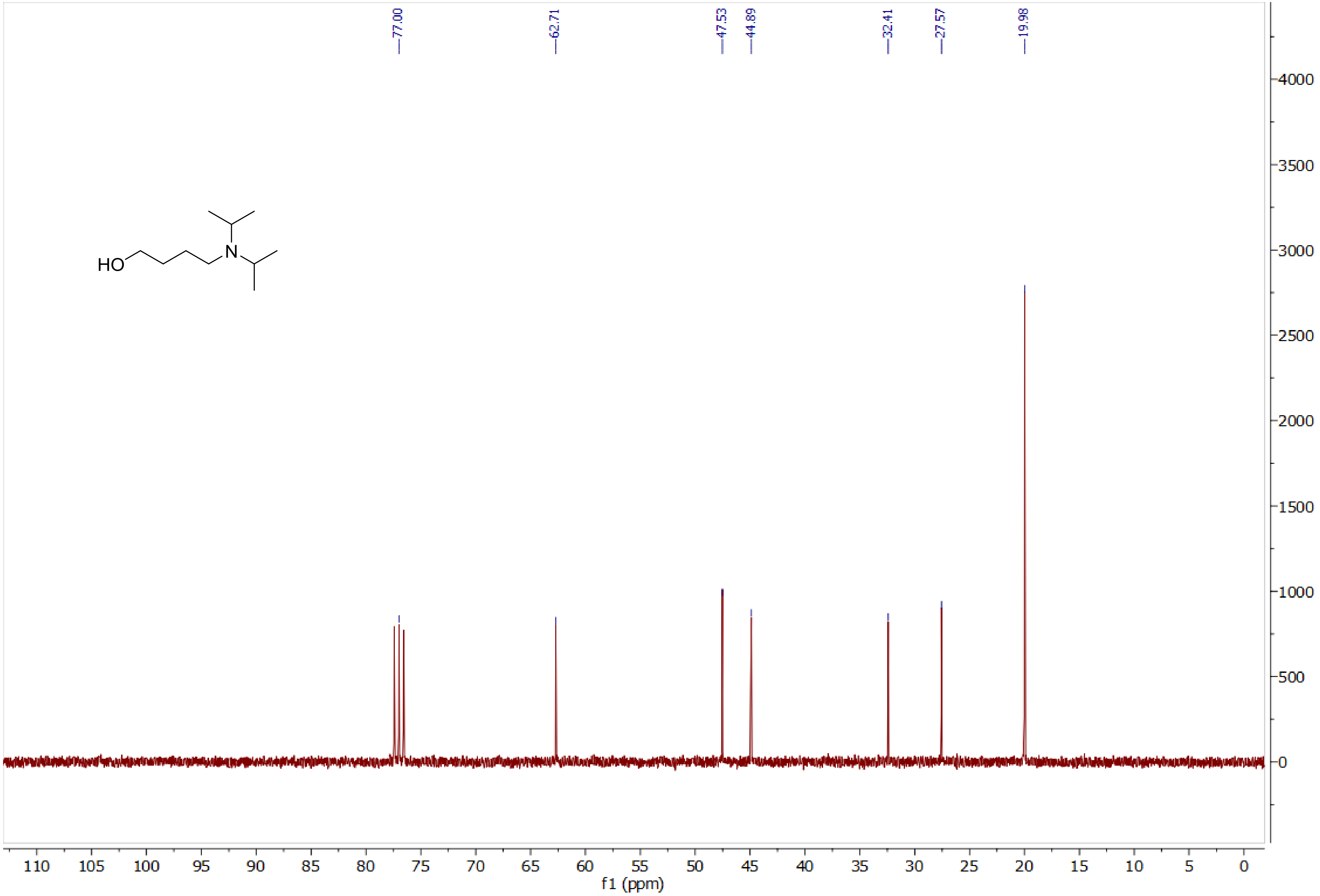

^{1}H NMR of 4-(*N*,*N*-diisopropylamino)-1,1-dimethoxybutane (**10**) (300 MHz, $CDCl_3$)

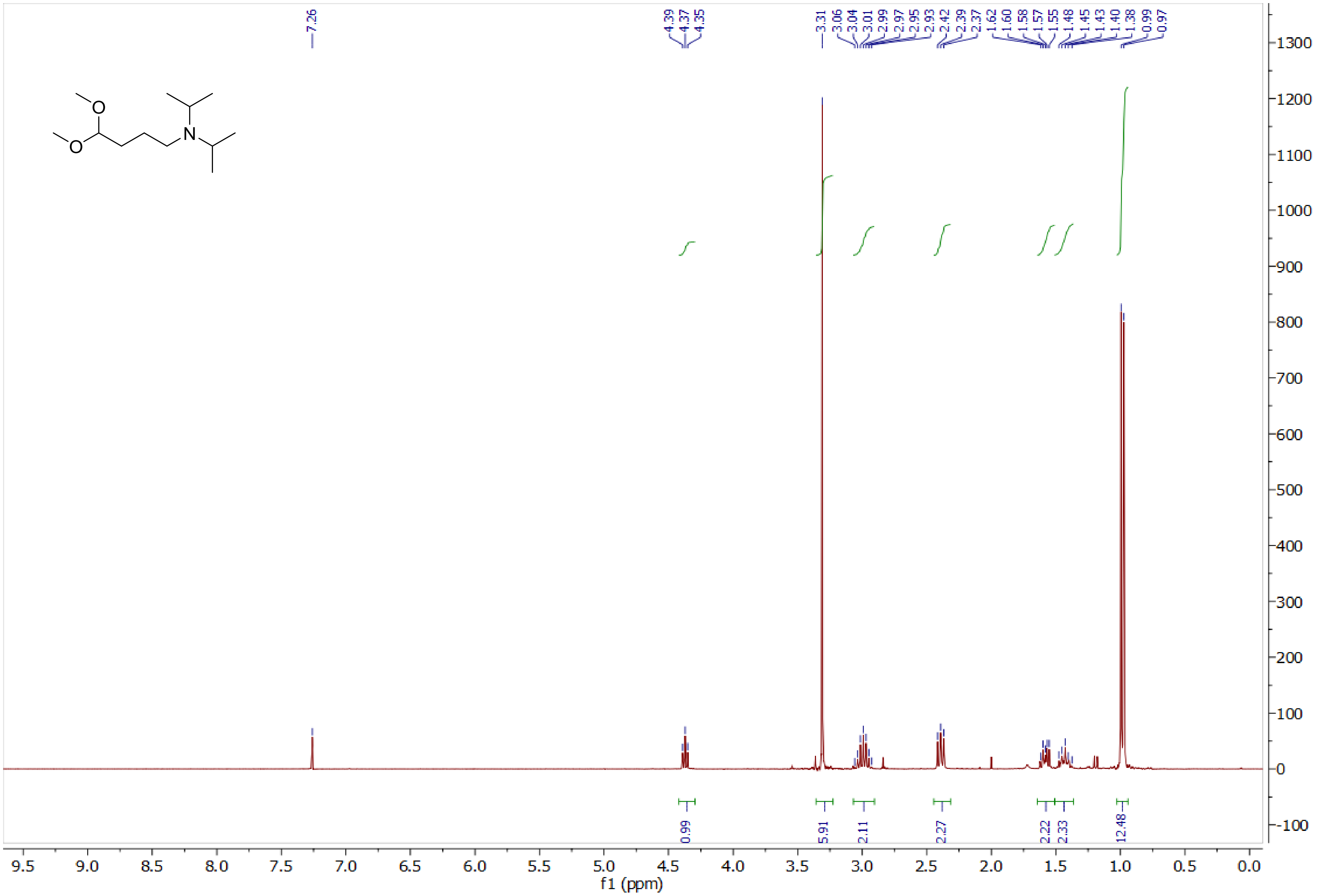

^{13}C NMR of 4-(*N*,*N*-diisopropylamino)-1,1-dimethoxybutane (**10**) (75 MHz, $CDCl_3$)

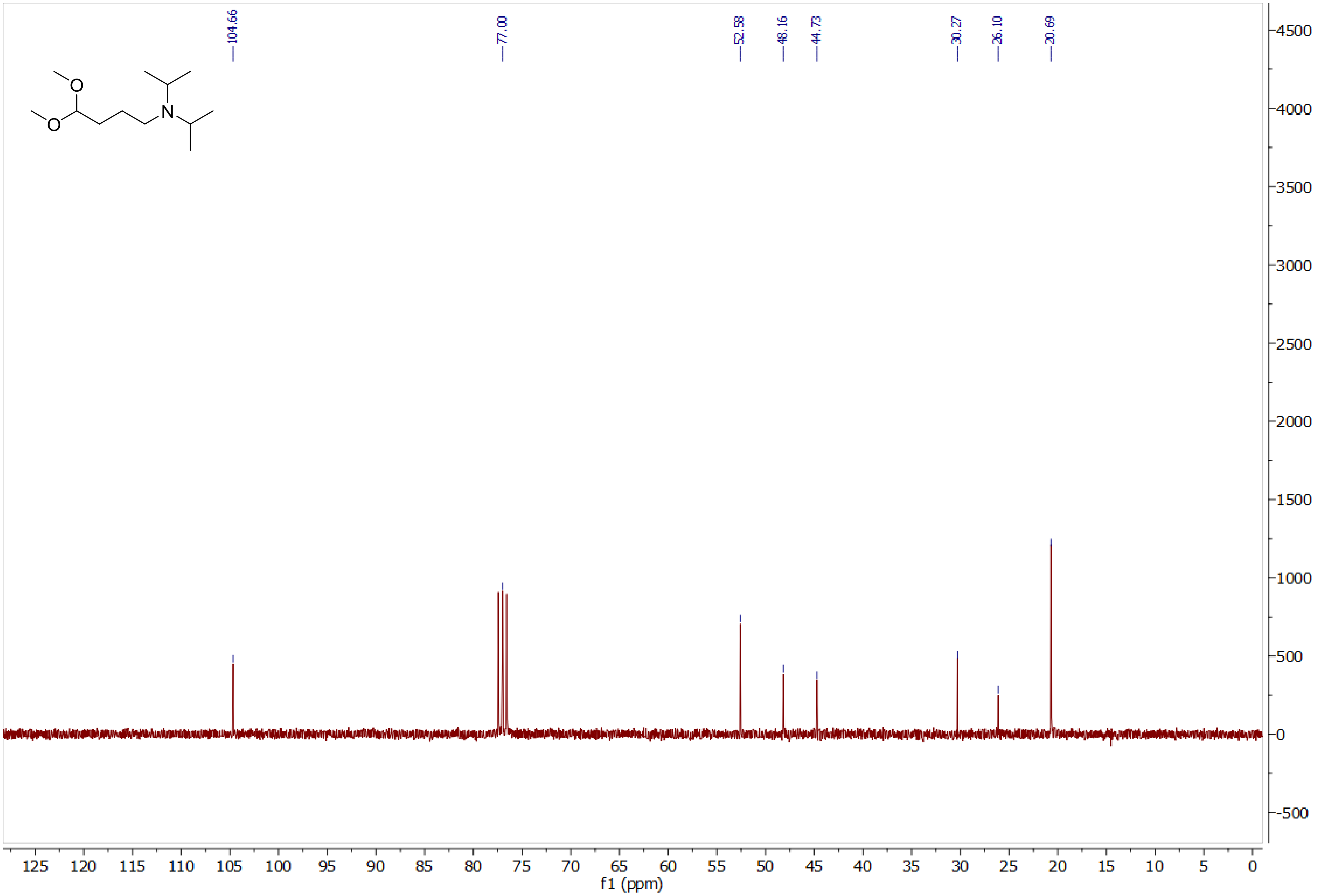

^{1}H NMR of 4-[*N*-[2-(*1H*-indol-3-yl)ethyl]-*N*-methyl]aminobutanoic acid hydrochloride (**1a**, **hapten I**) (300 MHz, DMSO-d$_6$)

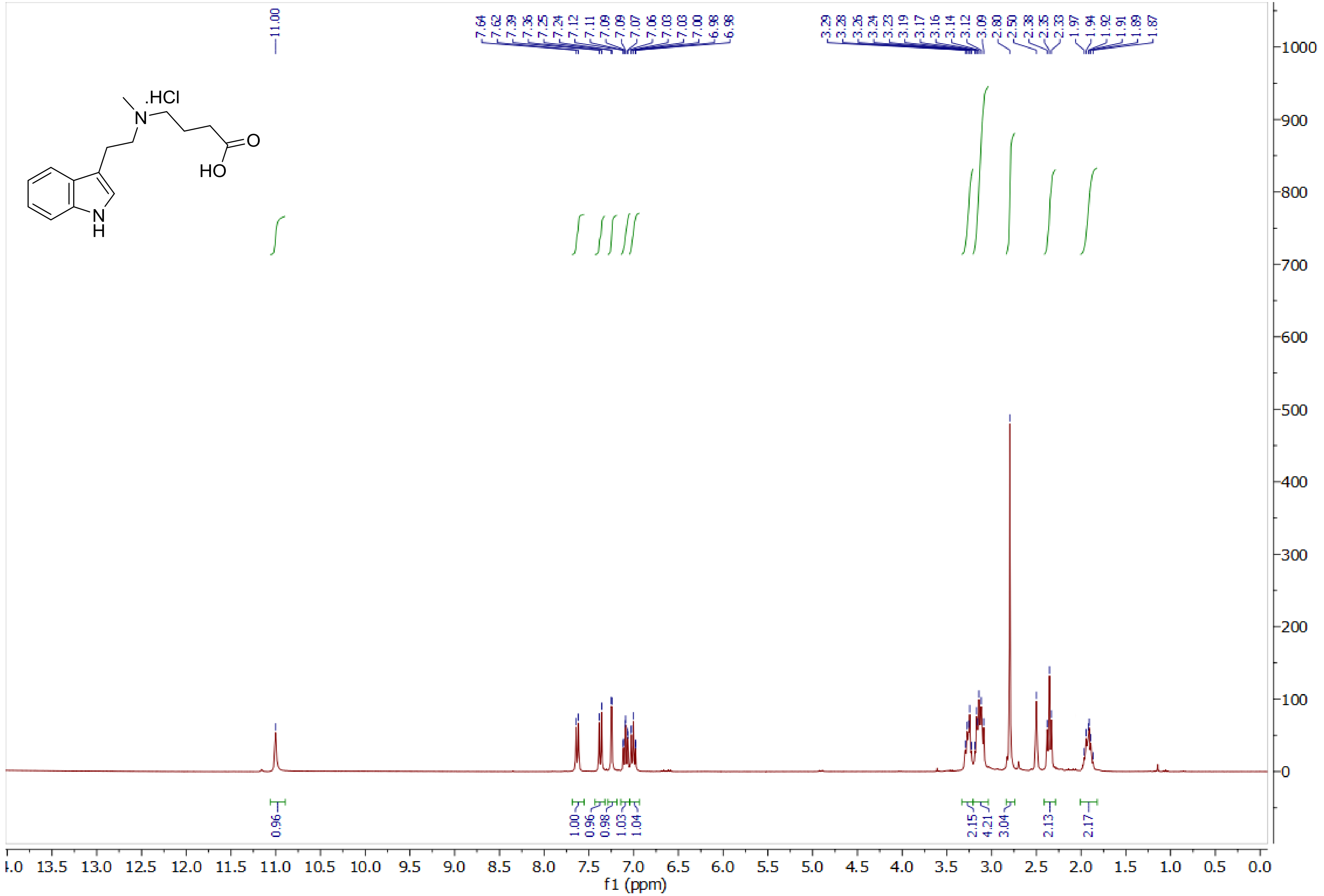

^{13}C NMR of 4-[*N*-[2-(*1H*-indol-3-yl)ethyl]-*N*-methyl]aminobutanoic acid hydrochloride (**1a**, **hapten I**) (75 MHz, DMSO-d_6)

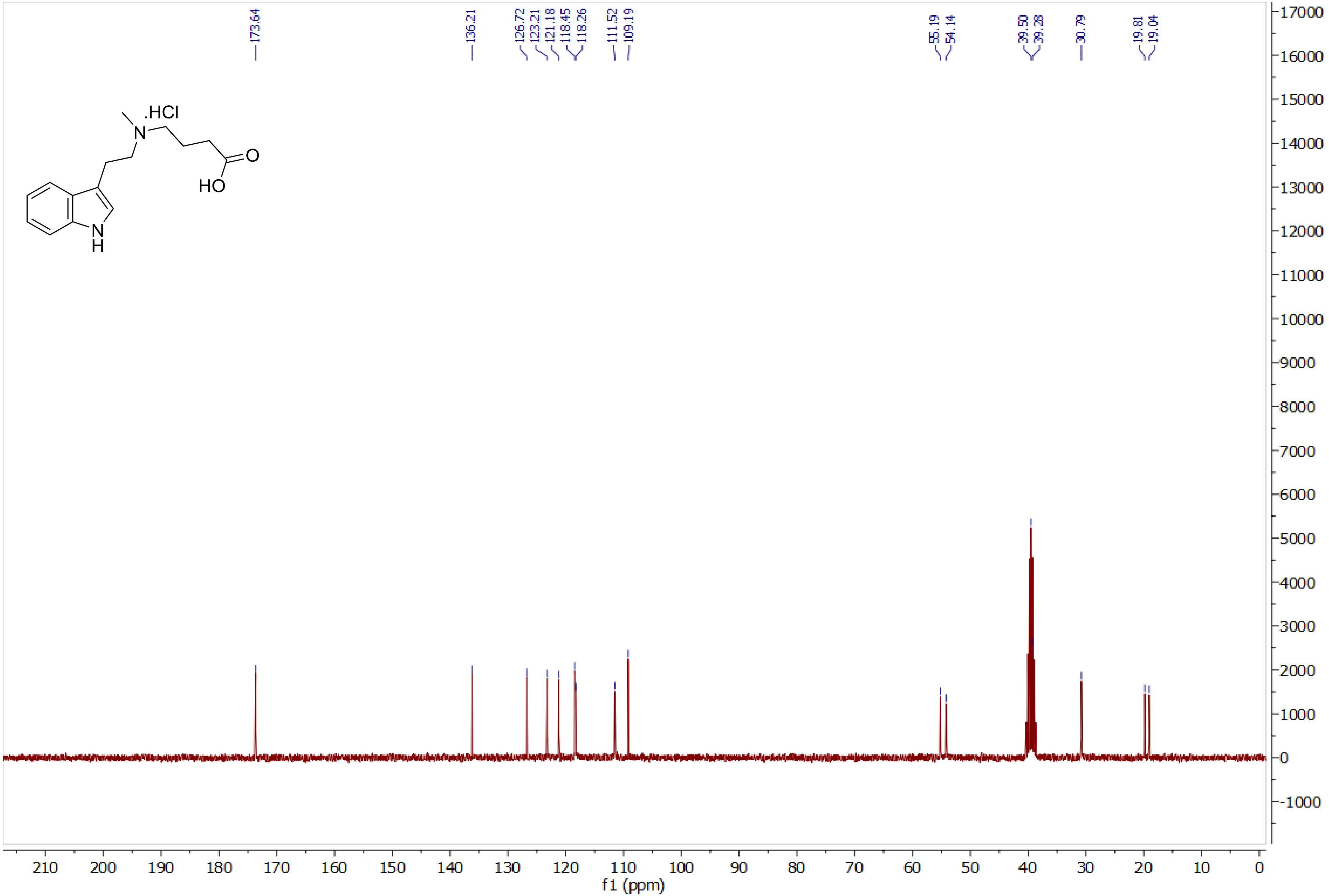

^{1}H NMR of 2-[3-[2-(*N*,*N*-dimethylamino)ethyl]-*1H*-indol-5-yl]acetic acid hydrochloride (**1b**, **hapten II**) (300 MHz, CD_3OD)

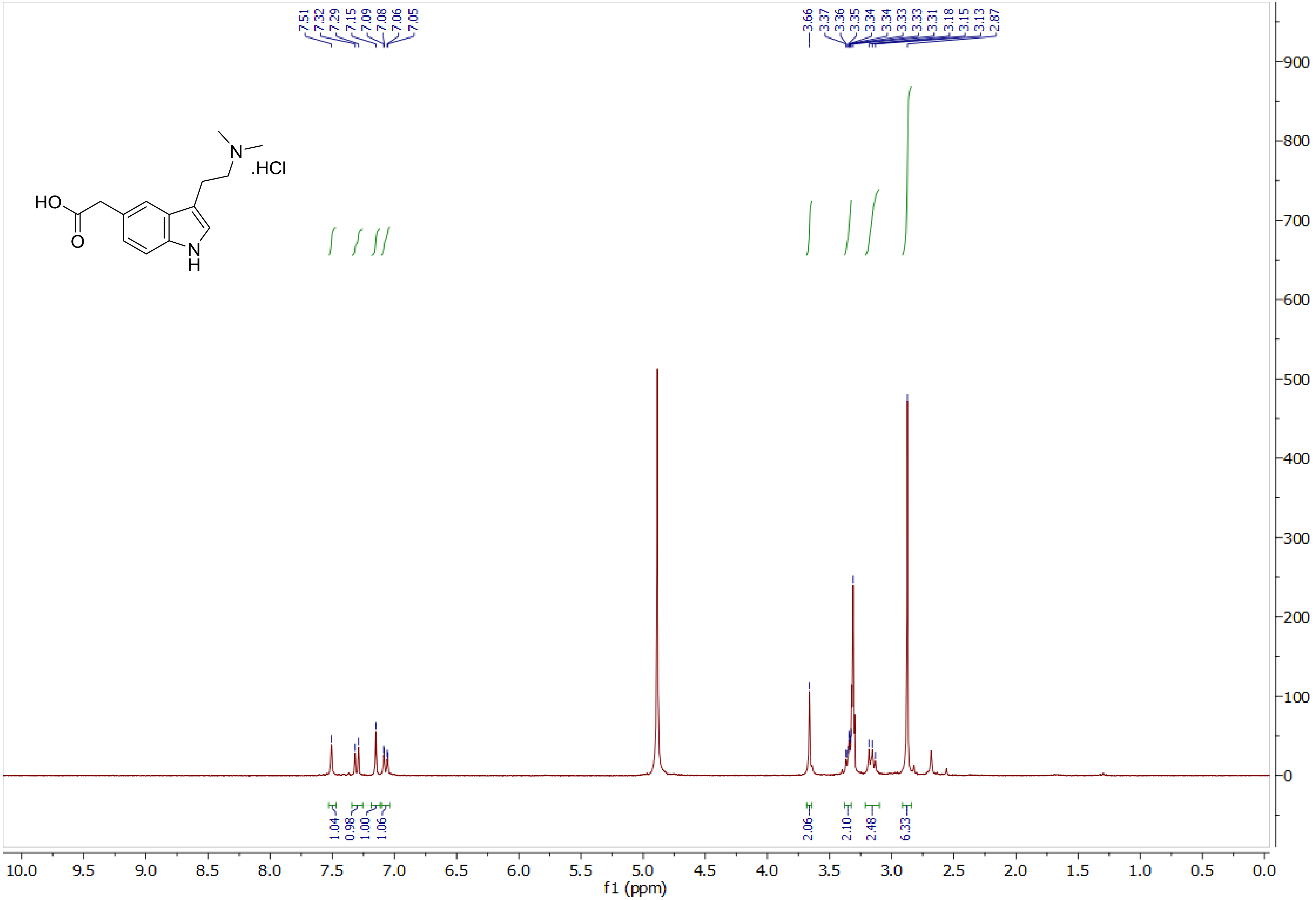

^{13}C NMR of 2-[3-[2-(*N*,*N*-dimethylamino)ethyl]-*1H*-indol-5-yl]acetic acid hydrochloride (**1b**, **hapten II**) (75 MHz, D_2O)

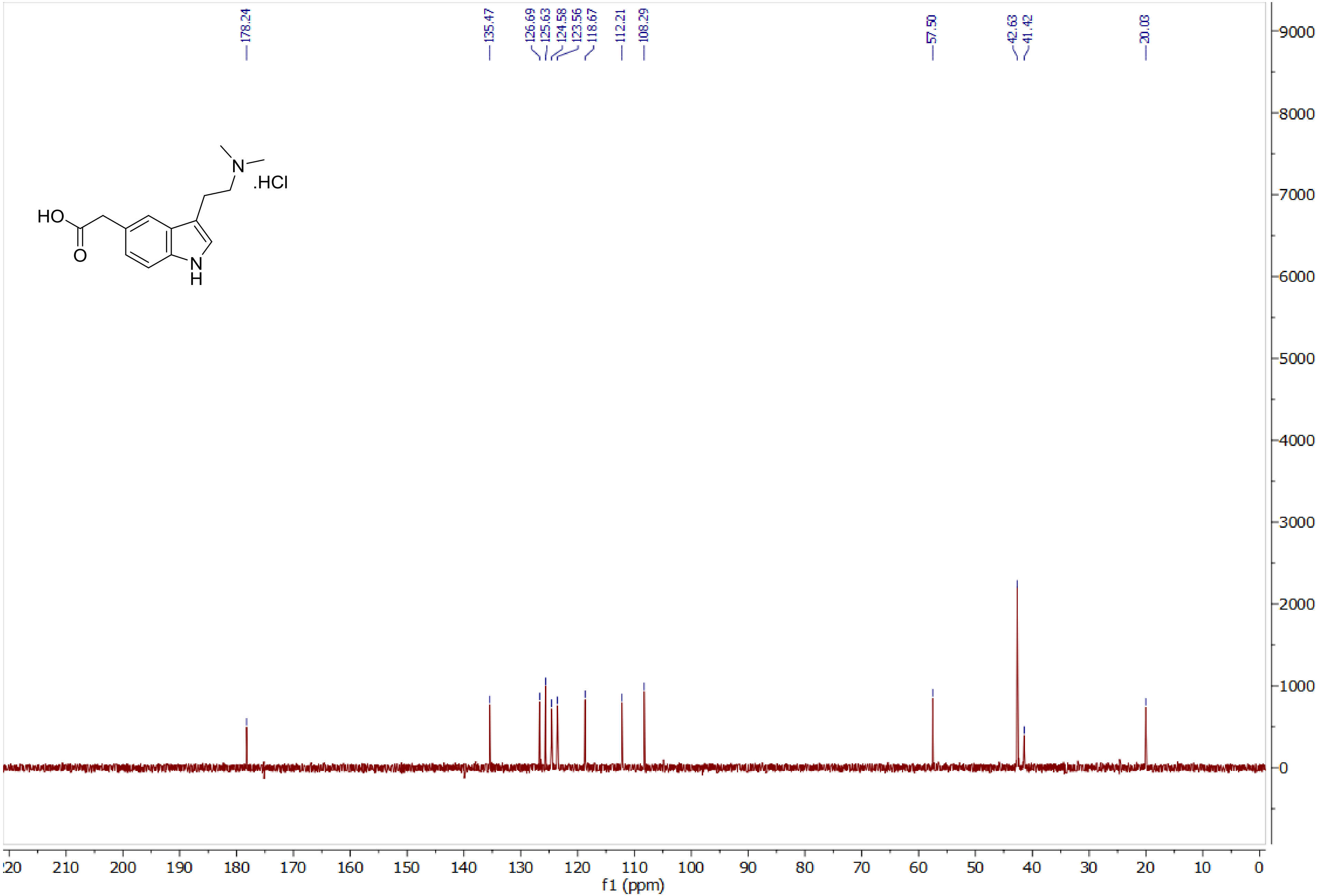

^{1}H NMR of 2-[3-[2-(*N*,*N*-dimethylamino)ethyl]-*1H*-indol-5-yloxy]acetic acid hydrochloride (**1c**, **hapten III**) (300 MHz, D_2O + DMSO-d_6)

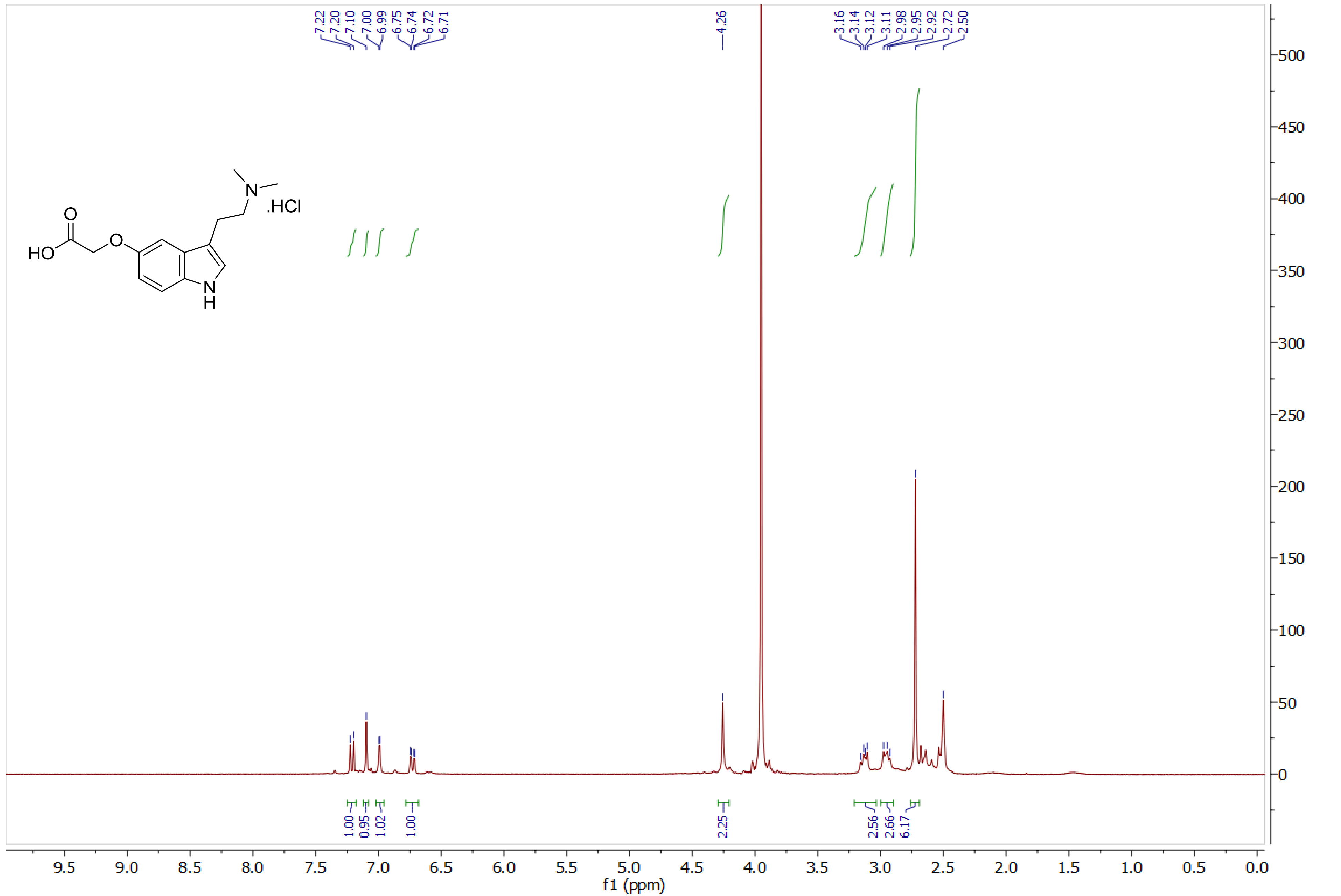

^{13}C NMR of 2-[3-[2-(*N*,*N*-dimethylamino)ethyl]-*1H*-indol-5-yloxy]acetic acid hydrochloride (**1c**, **hapten III**) (75 MHz, D_2O)

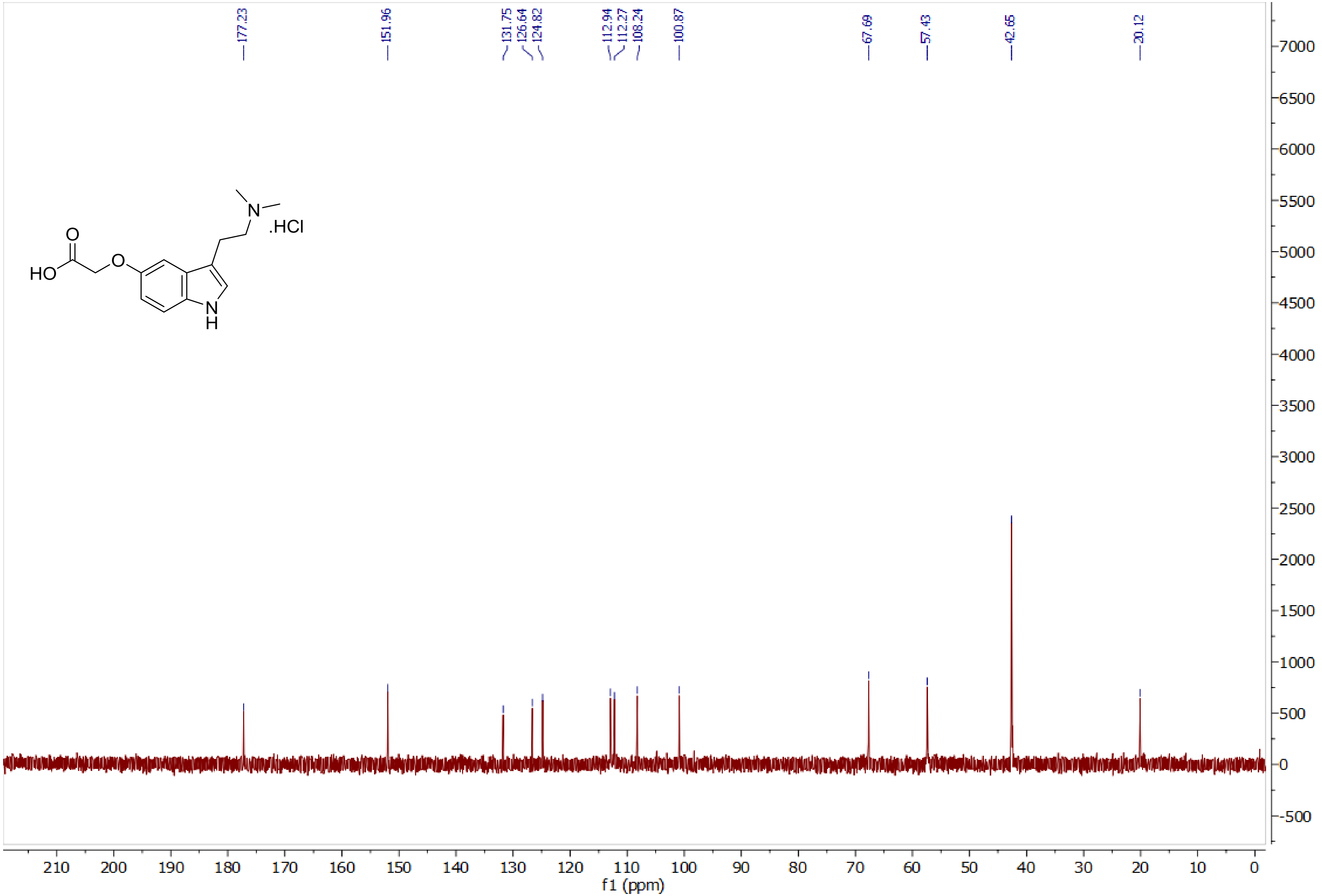

^{1}H NMR of -[3-[2-(*N*,*N*-diisopropylamino)ethyl]-*1H*-indol-5-yl]acetic acid hydrochloride (**1d, hapten IV**) (300 MHz, CD_3OD)

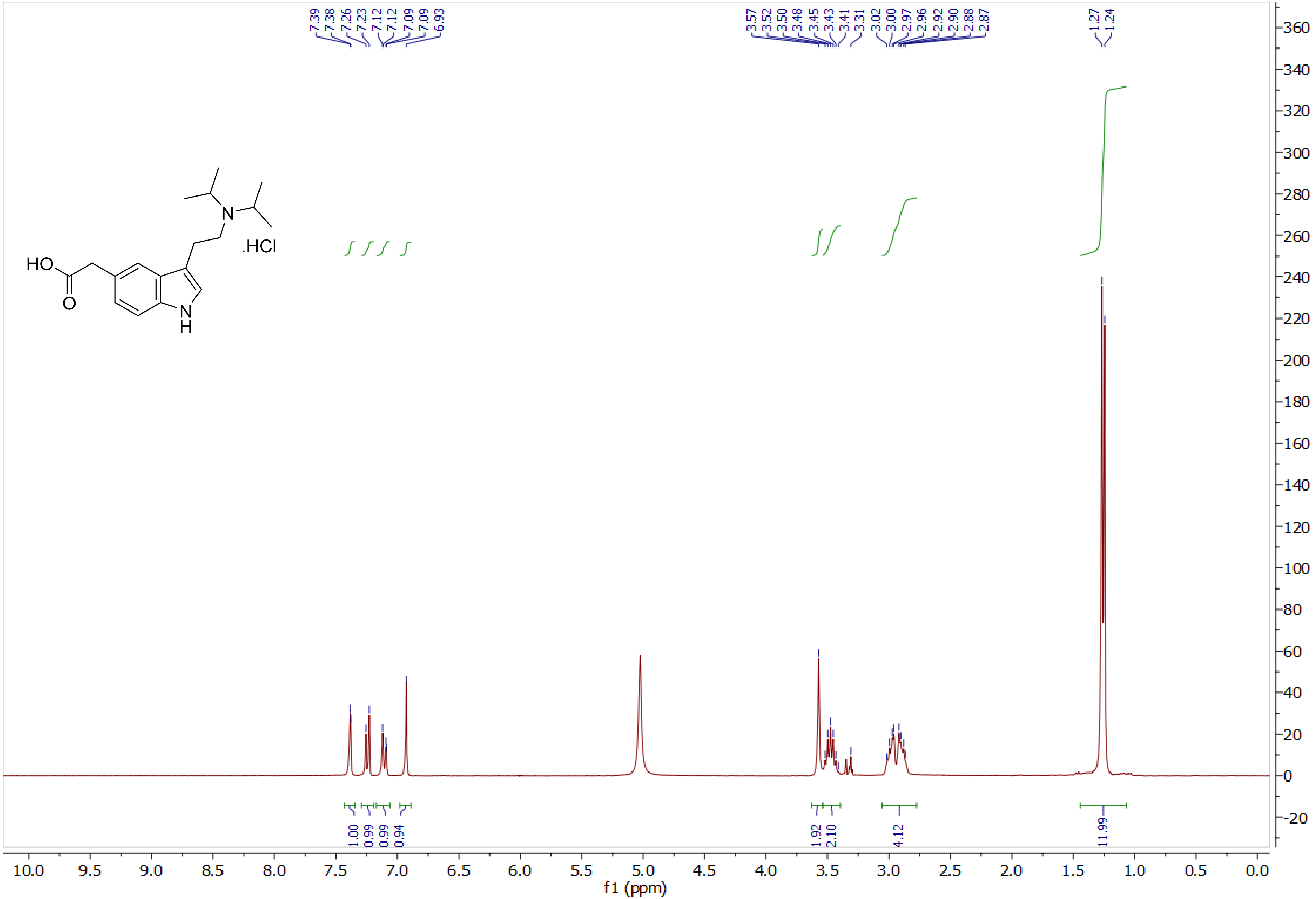

^{13}C APT NMR of -[3-[2-(*N*,*N*-diisopropylamino)ethyl]-*1H*-indol-5-yl]acetic acid hydrochloride (**1d**, **hapten IV**) (75 MHz, CD_3OD)

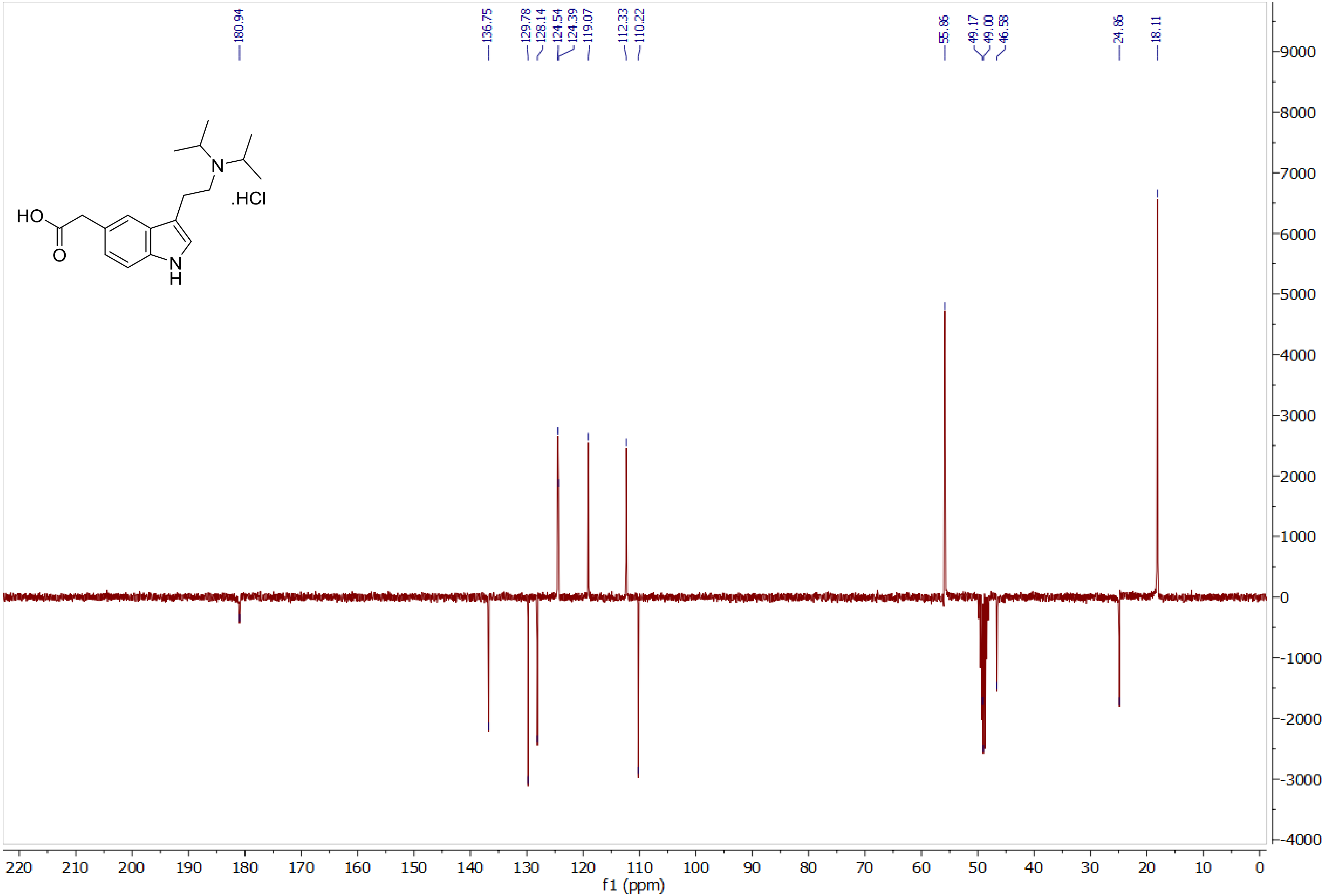

Supplement: RA-008-C8RA02528B-s001 [file RA-008-C8RA02528B-s001.pdf]
